# Distinct Fibroblast Lineages Give Rise to NG2+ Pericyte Populations in Mouse Skin Development and Repair

**DOI:** 10.3389/fcell.2021.675080

**Published:** 2021-05-28

**Authors:** Georgina Goss, Emanuel Rognoni, Vasiliki Salameti, Fiona M. Watt

**Affiliations:** Centre for Stem Cells and Regenerative Medicine, King’s College London, Guy’s Hospital, London, United Kingdom

**Keywords:** skin, dermis, fibroblast, pericyte, blood vessels, lineage tracing, wound healing

## Abstract

We have examined the developmental origins of Ng2+ perivascular cell populations that adhere to the basement membrane of blood vessels, and their contribution to wound healing. Neural/glial antigen 2 (Ng2) labeled most perivascular cells (70–80%) in developing and adult mouse back skin, a higher proportion than expressed by other pericyte markers Tbx18, Nestin and Pdgfrβ. In adult mouse back skin Ng2+ perivascular cells could be categorized into 4 populations based on whether they expressed Pdgfrα and Pdgfrβ individually or in combination or were Pdgfr-negative. Lineage tracing demonstrated that although Ng2+ cells in embryonic and neonatal back skin contributed to multiple cell types they did not give rise to interfollicular fibroblasts within the dermis. Lineage tracing of distinct fibroblast populations during skin development showed that papillary fibroblasts (Lrig1+) gave rise to Ng2+ perivascular cells in the upper dermis, whilst Ng2+ perivascular cells in the lower dermis were primarily derived from reticular Dlk1+ fibroblasts. Following wounding of adult skin, Ng2+ dermal cells only give rise to Ng2+ blood vessel associated cells and did not contribute to other fibroblast lineages. The relative abundance of Ng2+ Pdgfrβ+ perivascular populations was comparable in wounded and non-wounded skin, indicating that perivascular heterogeneity was maintained during full thickness skin repair. In the wound bed Ng2+ perivascular populations were primarily derived from Lrig1+ papillary or Dlk1+ reticular fibroblast lineages, according to the location of the regenerating blood vessels. We conclude that Ng2+ perivascular cells represent a heterogeneous lineage restricted population that is primarily recruited from the papillary or reticular fibroblast lineages during tissue regeneration.

## Summary Statement

NG2+ pericytes in healthy and wounded mouse skin originate from distinct fibroblast lineages and do not contribute to other dermal cell types during skin homeostasis or wound repair.

## Introduction

The blood vessel wall is composed of three layers, the intima, the media, and the adventitia (Tinajero and Gotlieb, 2020). The adventitia is the outermost layer of the blood vessel and represents a dynamic, heterogeneous cell compartment host to immune cells, endothelial cells, fibroblasts and pericytes (Majesky et al., 2011). Pericytes can be distinguished from other perivascular cell types by their prominent nucleus, minimal cytoplasm, and long cytoplasmic processes that are embedded within the basement membrane of blood vessels and make contact with underlying endothelial cells (Bergers and Song, 2005; Mills et al., 2013; Greenhalgh et al., 2015; Thomas et al., 2017). Communication between pericytes and endothelial cells is mediated via adhesion plaques that allow direct contact, and peg and socket contacts that facilitate paracrine signaling (Bergers and Song, 2005; Mills et al., 2013). The close interaction between pericytes and endothelial cells allows pericytes to regulate blood flow via vessel constriction, maintain vessel wall integrity, regulate vascular permeability, and control angiogenesis through directing endothelial cell proliferation and migration (Thomas et al., 2017).

Pericytes have been shown to express a range of different markers, individually and in combination (Armulik et al., 2011; Hsu et al., 2019). The most commonly used include Rgs5 (Bondjers et al., 2003), Pdgfrβ (Armulik et al., 2011), Nestin (Birbrair et al., 2013a), Tbx18 (Guimarães-Camboa et al., 2017), CD146 (Dvoretskiy et al., 2019), and Neural/glial antigen 2 (Ng2) (Armulik et al., 2011). However, none of these markers are pericyte specific as each marker can identify multiple dermal cell populations; for example Pdgfrβ is also expressed by adventitial fibroblasts (Stapor et al., 2014) and CD146 is also expressed by endothelial cells (Crisan et al., 2008; Guerrero-Juarez et al., 2019; Gay et al., 2020; Haensel et al., 2020). Amongst the markers Ng2 is the most widely documented because it consistently labels cells with pericyte features on multiple types of vasculature in different organs (Ozerdem et al., 2001; Ozerdem and Stallcup, 2004). Ng2 is a membrane spanning proteoglycan, also known as chondroitin sulfate proteoglycan 4 (CSPG4) and melanoma-associated chondroitin sulfate proteoglycan (MCSP), which can be membrane bound, or secreted and complexed with the extracellular matrix (ECM) (Stallcup, 2002). Functions attributed to Ng2 in various cell types include stimulating proliferation (Majumdar et al., 2003; You et al., 2014), increased cell spreading and motility (Majumdar et al., 2003; Fukushi et al., 2004; Ozerdem and Stallcup, 2004) and cell-cell communication (You et al., 2014). As well as being expressed by perivascular cell populations, in the skin Ng2 is expressed by other cell types, including keratinocytes (Kadoya et al., 2008; Armulik et al., 2011), adipocytes (Kadoya et al., 2008) and epidermal stem cells (Legg, 2003).

Mammalian skin comprises two layers: the epidermis and the underlying connective tissue dermis (Rognoni and Watt, 2018). At E16.5 mouse dorsal skin dermis begins to form distinct upper (papillary) and lower (reticular) layers (Driskell et al., 2013). The main cell type present in the dermis, fibroblasts, arises from a common progenitor that expresses Pdgfrα at E12.5 (Driskell et al., 2013). At E16.5 fibroblasts of the upper lineage express Blimp1, Lrig1, and Cd26, whilst those of the lower lineage express Dlk1 and Sca1 (Driskell et al., 2013). The different fibroblast lineages have distinct functions during skin development and regeneration (Driskell et al., 2013). The upper lineage (Lrig1+, Blimp1+) gives rise to the dermal papilla, dermal sheath and arrector pili muscle (Telerman et al., 2017). The lower lineage (Dlk1+) gives rise to reticular fibroblasts that deposit collagen and elastin rich extracellular matrix and to the preadipocytes (Cd24+) and adipocytes of the dermal white adipose tissue (DWAT; hypodermis) (Driskell et al., 2013; Jiang et al., 2018; Shook et al., 2018). Fibroblast heterogeneity is also a feature of human skin (Korosec et al., 2019; Shaw and Rognoni, 2020), although expression of marker genes is not well conserved across species (Philippeos et al., 2018). Pericytes are located on the vasculature throughout all layers of the dermis (Yamazaki et al., 2017). Although pericytes have been previously hypothesized to derive from adventitial fibroblasts based on observational data (Rhodin and Fujita, 1989) and are often considered related to fibroblast and smooth muscle cells (Stapor et al., 2014), their lineage relationship to skin fibroblast populations remains to be explored.

In the skin, perivascular cells including pericytes have been ascribed many important functions, from fibrosis to wound healing. In wound healing pericytes are reported to play roles in inflammation, angiogenesis, and re-formation of the epithelial barrier (Mills et al., 2013; Bodnar et al., 2016; Thomas et al., 2017). During the first stages of wound healing platelets release PDGF and TGF-β to promote pericyte migration, which results in the destabilization of the endothelial tube, stimulating endothelial migration and proliferation and facilitating neovascularization. Once nascent vasculature has been formed, pericytes promote vessel stabilization via paracrine signaling and cell-cell contact (Bodnar et al., 2016; Thomas et al., 2017). Following acute dermal or muscle injury, progeny from a Pdgfrα+ perivascular subpopulation of tissue-resident Adam12+ cells, which express pericyte markers (Ng2, Pdgfrβ) among other mesenchymal markers (Cd29, Cd44, Sca1), make up the majority of αSMA+ myofibroblasts (Dulauroy et al., 2012). These collagen producing αSMA+ myofibroblasts differentiate from tissue-resident Adam12+ cells within the perivascular space, which may include pericytes (Dulauroy et al., 2012; Greenhalgh et al., 2015). In a model of systemic sclerosis pericytes and myofibroblasts have been shown to display a phenotypic similarity in regards to ED-A FN and Thy-1 expression, fuelling the hypothesis of a pericyte to myofibroblast transition within the skin during microvascular damage (Rajkumar et al., 2005). In the lung, pericytes expressing Pdgfrβ and Ng2 transition into myofibroblasts when stimulated by TGF-β (Yamaguchi et al., 2020). Similarly in the kidney, Col1A1 expressing pericytes (Pdgfrβ+, Pdgfrα+, Cd73+) were identified as the major source of interstitial myofibroblasts within fibrosis (Lin et al., 2008; Chang et al., 2012), with TGF-β shown to trigger the pericyte to myofibroblast transition (Wu et al., 2013). In contrast, how pericyte populations contribute to tissue repair, and their relationship to other fibroblast populations in the skin, is largely unexplored. In particular it is not clear whether pericytes arise from common Pdgfrα+ fibroblast progenitors in E12.5 mouse back skin (Driskell et al., 2013).

Comparison of pericytes in multiple organs indicates that they have different lineage origins depending on tissue type and developmental stage. Pericytes from the face, brain and thymus derive from neural crest cells (Etchevers et al., 2001; Korn et al., 2002; Foster et al., 2008), whilst pericytes from the lung, liver and gut arise from the mesothelium (Wilm, 2005; Que et al., 2008; Asahina et al., 2011). In the skin, a recent study reported that a small proportion of pericytes is derived from the myeloid cell lineage (Yamazaki et al., 2017). Nevertheless, the lineage origin of the majority of skin pericytes and their tissue organization remains to be identified.

Using a combination of lineage tracing technology and immunostaining techniques we now demonstrate that multiple fibroblast lineages contribute to dermal Ng2+ perivascular populations in healthy mouse skin and following wounding. Once these perivascular cells (pericytes) acquire Ng2 expression they become lineage restricted and only contribute to blood vessel associated cell populations. Our findings reveal a previously unrecognized heterogeneity within the perivascular niche.

## Materials and Methods

### Mice

All experimental procedures were carried out under the terms of UK Home Office project license numbers PP70_8474 and PP0313918. All mice were maintained on a C57BL/6 background and experiments were conducted on mice of either sex from lines that had been generated previously: NG2DsRed (Jackson Laboratory, 008241), PdgfrαH2BEGFP (Hamilton et al., 2003), NG2CreER^*t*^ (Jackson Laboratory, 008538), Dlk1CreER^*t*^ (Driskell et al., 2013), Lrig1CreER (Page et al., 2013), and ROSA26-tdTomato mice (Jackson Laboratory, 007905).

Embryonic lineage tracing experiments utilized Ng2CreER^*t*^;tdTomato, Dlk1CreER^*t*^;ROSAtdTomato and Lrig1CreER;ROSAtdTomato mice in which tdTomato is expressed when the stop codon is removed via Tamoxifen inducible Cre-mediated recombination. Intraperitoneal injection of pregnant females with 150 μ*l* Tamoxifen (20 mg/ml) was conducted at embryonic day (E)18.5. Prior to injection tamoxifen was dissolved in corn oil and sonicated at 37°C for 20 min.

### Wound Healing

Wounding (2 mm diameter circular full thickness wounds) was performed as described previously (Rognoni et al., 2016) on adult mice between 8 and 14 weeks old when their hair cycle is in the telogen phase. For Ng2CreER^*t*^, Lrig1CreER, and Dlk1CreER^*t*^ lineage tracing post-wounding, Tamoxifen was injected at E18.5. Wounds were isolated from Ng2CreER^*t*^ mice at 4 days post wounding (DPW), 7DPW, and 10DPW. Wounds were isolated from Lrig1CreER and Dlk1CreER^*t*^ mice at 10DPW for histological analysis. The E18.5 time point for Tamoxifen treatment was selected because at this stage Lrig1 and Dlk1 are differentially expressed in the upper and lower dermis, respectively (Driskell et al., 2013).

### Histology and Microscopy

Tissue was collected at the indicated time points, fixed with 4% paraformaldehyde (PFA) for 20 min at room temperature and frozen embedded in optimal cutting temperature (OCT). Sections of 16 μm thickness or horizontal whole mounts of 60 μm were prepared. Sections of 16 μm thickness were further fixed with 4% PFA for 2 min, followed by PBS washes, blocking buffer and antibody incubation (Supplementary Table 1). Horizontal whole mounts were stained as described previously (Driskell et al., 2013) with antibodies listed in Supplementary Table 1. Briefly, staining was performed in 1.5 ml Eppendorf tubes with primary incubation overnight at 4°C and secondary incubation with DAPI at room temperature for 1 h. Samples were mounted using a small drop of 100% glycerol (Sigma). All microscopy was performed on a Nikon A1 upright confocal microscope and images were analyzed in Image J. Images displayed from horizontal whole mounts are maximum projection images generated from Z stacks in order to fully capture intact vascular networks. A minimum of 3 sections per mouse were analyzed.

### Flow Cytometry

Dermal fibroblasts were isolated as previously described (Jensen et al., 2010; Collins et al., 2011; Rognoni et al., 2018). The muscle and fat from dissected neonatal or adult back skin were scrapped off from the underside of the skin using a scalpel and the tissue was incubated overnight at 4∘C in a Dispase (Sigma) only solution. The epidermis was then carefully peeled off and discarded, leaving the intact dermis. The dermis was minced using a scalpel and enzymatically dissociated with a mixture of 1.25 mg/ml collagenase type 1 (Invitrogen), 0.5 mg/ml collagenase type 2 Worthington), 0.5 mg/ml collagenase type 1V (Sigma), 0.1 mg/ml hyaluronidase IVS (Sigma) and 50 U/ml DNase 1 for approximately 45 min at 37∘C. Dermal cell suspensions were passed through a 70 μm cell strainer and washed three times with PBS before being labeled with the following antibodies: Cd24 PerCP-Cy5.5 (eBioscience, Clone M1/69), Cd26 PerCP-Cy5.5 (eBioscience, Clone M194-112), Ly-6A/E APC (eBioscience, Clone D7), Cd140b APC (Thermo Fisher Scientific, 17-1402-82), Pref-1/Dlk1 APC (R&D Systems, FAB8634A), Lrig1 Alexa Fluor 488-conjugated (R&D Systems, FAB3688G). DAPI was used to exclude dead cells. FACS analysis was carried out with BD FACSCanto 1 or 11. Data analysis was performed using FlowJo software version 10.5.3.

### Quantification, Graphing, and Statistical Analysis

All graphs were generated using GraphPad Prism 8. Statistical significance was determined by Welch’s *t*-test. For identification of blood vessel pericyte and fibroblast co-location, the number of blood vessels in a sample was first quantified using Cd31 labeling in Image J software. Maximum projections of Z stack images were used to visualize the vascular network and allow quantification of branch points: each branch point was counted as a separate blood vessel. The number of Cd31 positive blood vessels that had pericytes expressing specific markers was then quantified and presented as a percentage of the total number of Cd31 positive blood vessels. For quantification of wound healing images, the region inside the wound bed was defined as the area flanked by the nearest hair follicles on both sides, extending from the base of the epidermis down to the muscle layer. Non-wounded areas were defined as areas distant from the wound bed either side of the wound.

At least 3 mice were analyzed for each condition. Adult mice were excluded from analysis if they had entered the anagen phase of the hair cycle or if the back skin was damaged (as a result of scratching or fighting).

## Results

### Ng2 Is Expressed by Most Perivascular Cells During Skin Development

Although a pericyte-specific marker has yet to be identified, Tbx18, Nestin, Pdgfrβ, CD146, and NG2 are all reported to label pericytes in multiple organs (Bondjers et al., 2003; Armulik et al., 2011; Birbrair et al., 2013a,b; Guimarães-Camboa et al., 2017). In order to identify the most widely expressed pericyte marker in mouse skin development and homeostasis we antibody labeled back skin sections with Tbx18, Nestin, Pdgfrβ, and NG2 during several developmental time points that coincided with rapid vasculature remodeling (Yamazaki et al., 2017). We also labeled adult skin during the resting (telogen) phase of the hair growth cycle when no angiogenesis takes place (Mecklenburg et al., 2000). Although CD146 is known to label pericytes it is also expressed by endothelial cells (Crisan et al., 2008; Guerrero-Juarez et al., 2019), which is a potential difficulty in accurately identifying cell type expression. Co-labeling with Cd31 (endothelial cell marker) allowed us to compare and analyze pericyte marker expression specifically on blood vessels in developing and adult skin. Since the abundance of blood vessels changes during development and also differs between the different dermal layers in adult skin we reported pericyte marker expression as the percentage of blood vessels with pericytes expressing each marker of interest.

**FIGURE 1:**
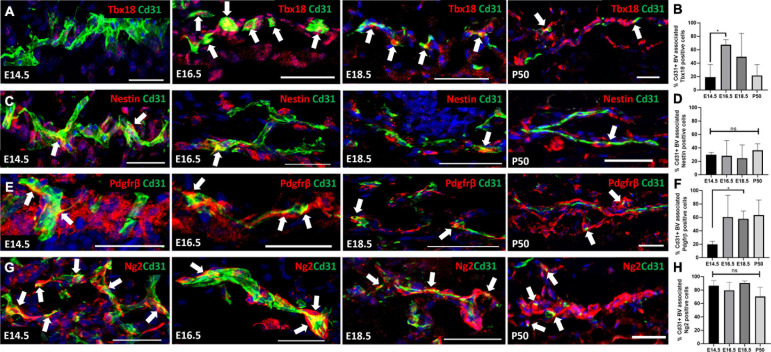
Expression of Tbx18, Nestin, Pdgfrβ, and Ng2 by perivascular cells in developing and adult skin. Sections of mouse back skin collected at the times shown were co-labelled with antibodies to Cd31 (green) and individual pericyte markers (red) and counterstained with DAPI (blue). White arrows highlight the presence of perivascular cells. Representative images of each time point are shown. The percentage of Cd31 positive blood vessels with each pericyte marker was calculated. **(A,B)** Tbx18, **(C,D)** Nestin, **(E,F)** Pdgfrβ, **(G,H)** Ng2. Error bars represent ±SD. *0.0152. *N=3* for each marker and developmental stage. Scale bars 50 μm.

At E14.5 and P50 approximately 20% of blood vessels had Tbx18 labeled associated cells, whilst at E16.5 the proportion was significantly higher (70%) (Figures 1A,B). The proportion of Nestin+ blood vessel associated cells was approximately 20–30% at all-time points analyzed (Figures 1C,D). In addition, Nestin+ dermal cells were present in the vicinity of blood vessels at E16.5 (Figure 1C). At E14.5 approximately 20% of blood vessels had associated Pdgfrβ+ cells (Figures 1E,F). At E16.5, E18.5 and P50 this rose to approximately 60% of vessels, although the increase was only statistically significant at E18.5 due to high variability at the E16.5 and P50 time-points (Figures 1E,F). In contrast to Tbx18, Nestin and Pdgfrβ, Ng2 labeled a high percentage (70–80%) of blood vessel associated cells at all-time points investigated (Figures 1G,H). These findings demonstrate the heterogeneous nature of perivascular cells during skin development and led us to select Ng2 as the pericyte marker for lineage tracing studies due its consistent expression on the majority of blood vessels, regardless of developmental stage.

We employed the well-established Ng2DsRed reporter mouse (Birbrair et al., 2013a, 2014a, b; Nirwane et al., 2017; Zhao et al., 2018), in which cells express the optimized red fluorescent protein variant DsRed.T1 under the control of the Ng2 promoter (Jackson Lab, 008241), for further studies. Antibody labeling of sections of skin from the Ng2DsRed reporter mouse confirmed over 90% co-localisation of anti-Ng2 and DsRed in perivascular cells (Supplementary Figure 1). Throughout postnatal development and in adult skin Ng2 was consistently expressed by cells associated with micro-vessels in the papillary and reticular dermis as well as large blood vessels in the reticular and DWAT layer (Supplementary Figure 2).

Although Ng2 was expressed by a high percentage of perivascular cells within the skin it was not specific to blood vessel associated cells. Other Ng2DsRed+ cells within the dermis included the dermal papilla (Supplementary Figures 2C,D), the arrector pili muscle (APM) (Supplementary Figure 3A), the dermal sheath of hair follicles (Supplementary Figure 3B) and keratinocytes within the lower proximal cup of the hair follicle (HF) bulge (Supplementary Figure 3C). Ng2DsRed+ cells that co-expressed Nephronectin were detected at the anchor point of the APM to the HF bulge (Supplementary Figure 4A) and Ng2DsRed+ cells co-expressing Egfl6 were present in the bulge itself (Supplementary Figure 4B), suggesting a possible contribution to the APM niche (Fujiwara et al., 2011). Despite Ng2 being expressed by multiple cell types, Ng2+ perivascular cells in the dermis could readily be distinguished by their location on the blood vessels.

### Four Distinct Ng2+ Perivascular Populations Are Present in Adult Mouse Back Skin

**FIGURE 2:**
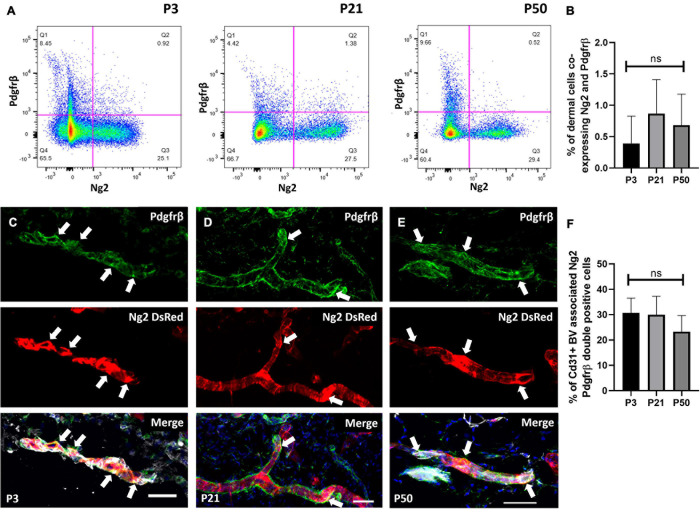
A population of perivascular cells expresses Ng2 and Pdgfrβ. **(A)** Flow cytometry of Ng2DsRed adult mouse back dermal cells labeled with Pdgfrβ at P3, P21, and P50. **(B)** The percentage of live dermal cells co-expressing Pdgfrβ and Ng2 at P3, P21, and P50. **(C–E)** Immunostaining of Ng2DsRed mouse back skin with anti-Pdgfrβ (green) and Cd31 (grey) at P3 **(C)**, P21 **(D)**, and P50 **(E)** showing significant co-expression of both markers (white arrows) by pericyte populations. *N* = 3. **(F)** Graph depicting the percentage of Cd31 positive blood vessel associated cells expressing both Ng2 and Pdgfrβ. Scale bars 50 μm. Flow cytometry *N* = 4. Statistical test: One-way ANOVA. Error bars represent ±SD.

We next investigated whether dermal perivascular cells co-expressed Ng2 and Pdgfrα or Pdgfrβ. Pdgfrβ is an abundantly expressed pericyte marker (Figures 1E,F) and Pdgfrα is of interest as it is expressed by both upper and lower fibroblast lineages at all stages of mouse dermal development (Driskell et al., 2013). It has been reported that pericytes at the tip of new blood vessels in healing skin wounds are Pdgfrβ+NG2- and those at the rear (i.e., more mature) are Pdgfrβ+ NG2+ (Morikawa and Ezaki, 2011). Utilizing Ng2DsRed reporter mice and Pdgfrβ antibody labeling in anagen mouse back skin at P3 and telogen mouse back skin at P21 and P50, flow cytometry of mouse dermal cells showed approximately 1% of Ng2+ cells co-expressed Pdgfrβ (Figures 2A–D). Single cell RNA sequencing studies have demonstrated that perivascular and endothelial cells account for approximately 1% of all isolated dermal cells at anagen and 20% at telogen (Joost et al., 2020). Quantifying specifically the expression of perivascular cells by immunostaining of Ng2DsRed mouse back skin with anti-Pdgfrβ at P3, P21 and P50 revealed that 25–30% of perivascular cells were Ng2 Pdgfrβ double positive (Figures 2C–F).

We next investigated whether dermal perivascular cells in mouse back skin co-expressed Ng2 and Pdgfrα. Pdgfrα has previously been reported to be expressed by perivascular adventitial fibroblasts in the tunica adventitia layer of blood vessels, which are often found adjacent to pericytes and endothelial cells (Guimarães-Camboa and Evans, 2017). Pdgfrα is also expressed in a subset of perivascular Adam12+ cells which become activated upon dermal and muscle injury to contribute to tissue repair (Dulauroy et al., 2012).

**FIGURE 3:**
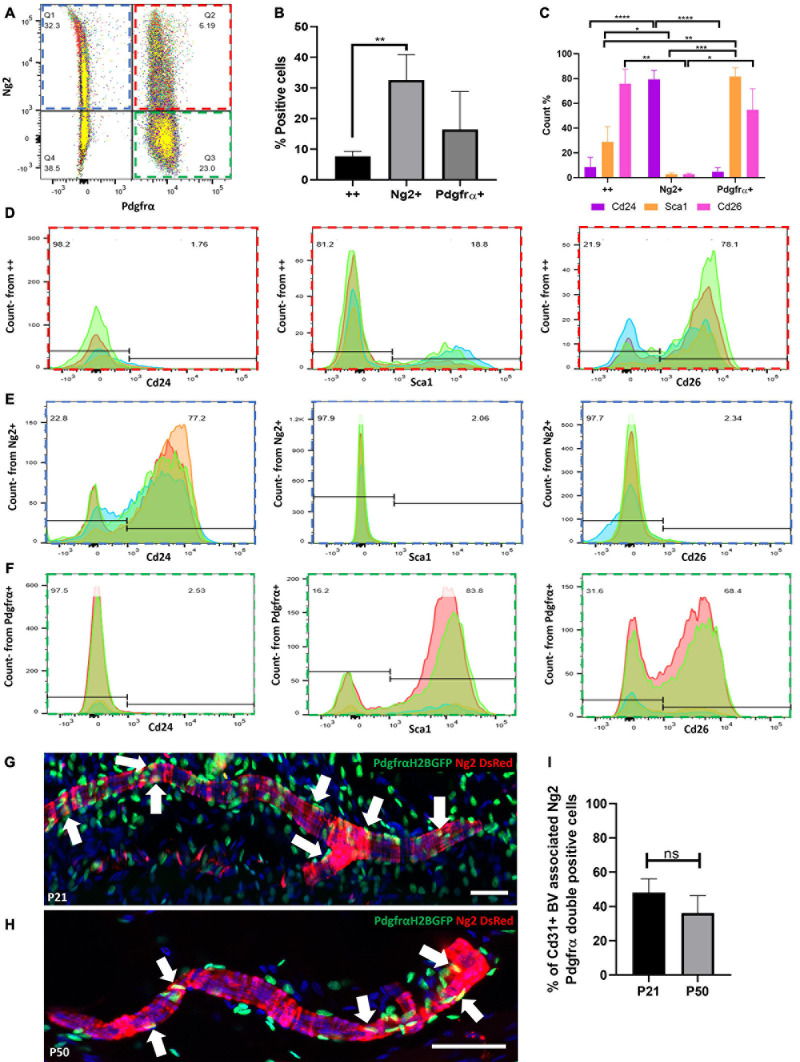
Expression of Ng2 and Pdgfrα in dermal cell populations reveals a significant population of Ng2+Pdgfrα+ perivascular cells. **(A)** Pool of 4 flow cytometry plots of PdgfrαH2BGFP; Ng2DsRed double transgenic adult mouse back dermal cells. Approximately 6% of dermal cells expressed both Ng2 and Pdgfrα. **(B)** The percentage of live dermal cells expressing Pdgfrα and Ng2 individually or in (++). **(C)** Graph depicting the percentage of cells expressing Cd24, Sca1, and Cd26 within Ng2+ Pdgfrα+, Ng2+ Pdgfrα−, or Ng2− Pdgfrα+ dermal populations corresponding to **(D–F)**, respectively. **(D–F)** Flow cytometry panels showing Ng2 and Pdgfrα expressing dermal cell populations **(D)** Ng2+ Pdgfra+ (red), **(E)** Ng2+ Pdgfra- (blue), **(F)** Ng2- Pdgfra+ (green) which co-expressed Cd24, Sca1, or Cd26. **(G,H)** Immunostaining of PdgfrαH2BGFP; Ng2DsRed double transgenic mice at P21 **(G)** and P50 **(H)** identifying a proportion of perivascular cells expressing both Ng2 and Pdgfrα (white arrows). Representative images shown. **(I)** Graph depicting the percentage of blood vessel associated cells which co-expressed Ng2 and Pdgfrα. *P≤0.05, **P≤0.01, ***P≤0.001, and ****P≤0.0001. *N* = 5 for each flow cytometry experiment and immunostaining experiment. Scale bars 100 μm. Error bars represent ±SD. Statistical test: One-way or two-way ANOVA.

Flow cytometry of dermal cells isolated from double transgenic mice expressing Ng2DsRed and PdgfrαH2BEGFP (enhanced GFP fused to histone H2B expressed via the Pdgfrα promoter) (Hamilton et al., 2003; Collins et al., 2011) at P21 showed that approximately 6% of dermal cells co-expressed Ng2 and Pdgfrα (Figures 3A,B). In addition to pericytes and adventitial fibroblasts, this double positive population is likely to include fibroblasts from the APM, the dermal sheath and the dermal papilla (Supplementary Figures 2, 3). Flow cytometry further revealed that the double positive population expressed high levels of two other fibroblast markers, Cd26 (Dpp4, which labels reticular fibroblasts in adult dermis and papillary cells in developing dermis) and Sca1 (also known as Ly6a, which labels fibroblasts of the reticular lineage) (Driskell et al., 2013; Rinkevich et al., 2015). Cd26 was expressed at a much higher level than Sca1 (Figures 3C,D). In contrast, Ng2+Pdgfrα- cells predominantly expressed the preadipocyte marker Cd24 (Figure 3E), consistent with the observation that Ng2−/− mice have reduced DWAT (Kadoya et al., 2008). The Pdgfrα+Ng2− population expressed minimal Cd24 and high levels of Sca1 and Cd26, thus representing the majority of interfollicular dermal fibroblasts (Figure 3F).

To discover whether Ng2 and Pdgfrα were co-expressed by the same population of perivascular cells, immunofluorescence staining of mouse back skin was conducted at P21 and P50 (Figures 3G,H) and the percentage of Cd31 blood vessels with associated cells expressing Ng2 and Pdgfrα was quantified (Figure 3I). Approximately 50% of perivascular cells co-expressed Ng2 and Pdgfrα at P21, whilst this decreased to approximately 35% at P50 (Figure 3I). These results demonstrate that besides Ng2+ Pdgfrβ+/− perivascular populations there is a previously unrecognized Ng2+ Pdgfrα+ perivascular population.

**FIGURE 4:**
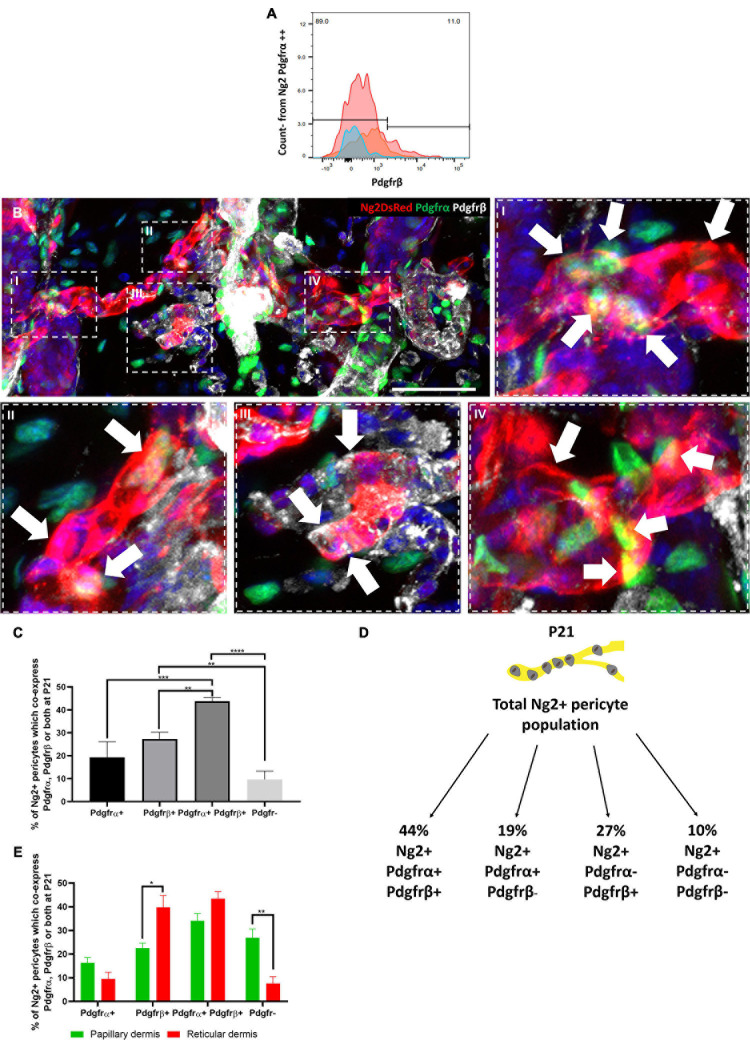
Four distinct Ng2 perivascular populations are present in adult mouse back skin based on Pdgfrα and/or Pdgfrβ co-expression. **(A)** Pool of 3 flow cytometry plots of PdgfrαH2BGFP; Ng2DsRed double transgenic adult mouse back dermal cells at P21. Live dermal cells co-expressing Ng2 and Pdgfrα were assessed for Pdgfrβ expression. **(B)** Immunofluorescence of a dermal blood vessel from PdgfrαH2BGFP; Ng2DsRed double transgenic mouse labelled with anti-Pdgfrβ (gray). High magnification images revealed the presence of 4 Ng2 perivascular populations. Ng2+ Pdgfrα– Pdgfrβ– cells can be seen in all high magnification panels. Arrows show I, II: Ng2+ Pdgfrα+ Pdgfrβ+, III: Ng2+ Pdgfrα- Pdgfrβ+, IV: Ng2+ Pdgfrα+ Pdgfrβ– Representative images shown. **(C)** Graph depicting the percentage of Ng2+ perivascular cells which are Pdgfr-, Pdgfrα– Pdgfrβ+, Pdgfrα+ Pdgfrβ−, and Pdgfrα+ Pdgfrβ+ at P21. **(D)** Schematic illustrating the percentage contributions of each Ng2+ perivascular subpopulation to the total Ng2+ perivascular population present at P21. **(E)** Graph depicting the percentage of Ng2+ perivascular cells which express Pdgfrα– Pdgfrβ+, Pdgfrα– Pdgfrβ+ and Pdgfrα+ Pdgfrβ+ in papillary and reticular layers of the dermis at P21. *P≤0.05, **P≤0.01, ***P≤0.001, and ****P≤0.0001. *N* = 3 Scale bars 100 μm. Error bars represent ±SD. Statistical analysis conducted using One-way ANOVA or Multiple *T*-Tests using the Holm-Sidak method.

To investigate whether Ng2+ Pdgfrα+ and Ng2+ Pdgfrβ+ populations represent distinct dermal populations, we isolated P21 dermal cells from Ng2DsRed; PdgfrαH2BEGFP double transgenic mice and performed flow cytometry with anti-Pdgfrβ. This revealed that approximately 11% (*N* = 3, ±SD: 0.74) of dermal Ng2 Pdgfrα double positive cells also expressed Pdgfrβ (Figure 4A).

To determine the proportions of the different Ng2+ perivascular populations immunofluorescence staining of P21 double transgenic mouse back skin with anti-Pdgfrβ was conducted. This revealed the presence of four Ng2+ perivascular populations: Ng2+ Pdgfrα+ Pdgfrβ+, Ng2+ Pdgfrα+ Pdgfrβ−, Ng2+ Pdgfrα− Pdgfrβ+, and Ng2+ Pdgfrα−Pdgfrβ− (Figures 4B–D). 44% of Ng2+ blood vessel associated cells co-expressed Pdgfrα and Pdgfrβ, whilst 19% expressed Pdgfrα but not Pdgfrβ, and 27% expressed Pdgfrβ but not Pdgfrα (Figures 4C,D). Approximately 10% of Ng2+ perivascular cells did not express Pdgfrα or Pdgfrβ (Figure 4D). All 4 populations were present in both papillary and reticular layers of the dermis (Figure 4E). However, there were significantly more Ng2+ Pdgfrα− Pdgfrβ+ cells in the reticular than the papillary layer, whereas there were significantly more Ng2+ Pdgfr− cells in the papillary compared to the reticular layer (Figure 4E). The abundance of the other perivascular subpopulations within the dermis was similar in both layers (Figure 4E).

In summary, based on Pdgfrα and Pdgfrβ expression we have identified four previously uncharacterised Ng2 expressing perivascular populations in adult skin, revealing unprecedented heterogeneity within the perivascular niche.

### During Skin Development Ng2+ Dermal Cells Contribute to Pdgfrβ+ and Pdgfrα+ Perivascular Cells

**FIGURE 5:**
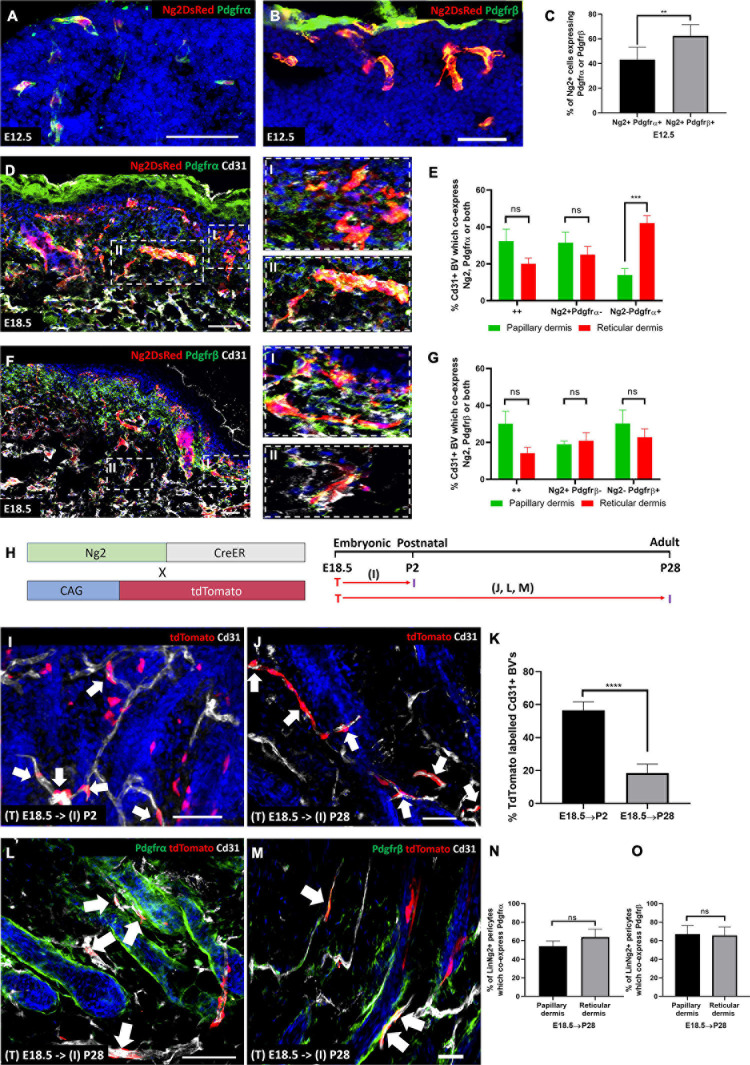
During skin development Ng2+ dermal cells contribute to Pdgfrα/β perivascular populations. **(A,B)** Ng2 DsRed mouse back skin at E12.5 labeled with Pdgfrα **(A)** or Pdgfrβ **(B)**. **(C)** Graph depicting the percentage of Ng2+ perivascular cells expressing Pdgfrα/β at E12.5. **(D)** Ng2 DsRed mouse back skin at E18.5 labeled with Pdgfrα (green) and Cd31 (gray). Panels on the righthand side are higher magnifications of papillary (I) and reticular (II) blood vessels. **(E)** The percentage of Cd31+ blood vessels in the papillary and reticular layers of the dermis which express Ng2 Pdgfrα populations. **(F)** Ng2 DsRed back skin at E18.5 labeled with Pdgfrβ (green) and Cd31 (gray). Panels on the righthand side are higher magnifications of papillary (I) and reticular (II) blood vessels. **(G)** The percentage of Cd31+ papillary and reticular blood vessels with Ng2 Pdgfrβ populations. **(H)** Ng2CreER labeling strategy and timing of Tamoxifen labeling (T) and subsequent tissue isolation **(I)**. Panels corresponding to labeling and isolation are highlighted. **(I,K)** Immunostaining of Ng2CreER; tdTomato mice with anti-Cd31 (gray) and DAPI nuclear counterstain (blue) labeled at E18.5 and isolated at P2 **(I)** and P28 **(J)**. Ng2 tdTomato expression by perivascular cells are highlighted with white arrows. **(K)** Graph depicting the percentage of tdTomato labeled blood vessel associated cells at P2 and P28. **(L,M)** Immunostaining of Ng2CreER; tdTomato mice induced at E18.5 and isolated at P28 labeled with Pdgfrα **(L)** or Pdgfrβ **(M)** and Cd31. **(N,O)** Graphs depicting the percentage of Ng2 tdTomato labeled perivascular cells which co-express Pdgfrα **(N)** or Pdgfrβ **(O)** in papillary and reticular layers of the dermis. Statistical test: Paired *T*-Test. Error bars represent ±SD. **P≤0.01, ***P≤0.001, and ****P≤0.0001. *N* = 3 Scale bars 50 μm. Representative images of the immunostaining are shown.

Prior to performing lineage tracing of Ng2+ cells in skin development and homeostasis, we conducted immunostaining of Ng2DsRed mice at E12.5 with Pdgfrα and Pdgfrβ (Figures 5A,B). E12.5 is of interest as this is when the Pdgfrα+ common fibroblast progenitor is found within developing skin (Driskell et al., 2013). At E12.5 Ng2+ Pdgfrα+ and Ng2+ Pdgfrβ+ double positive cells were both detected in the developing dermis (Figures 5A,B). However, the percentage of Ng2+ cells that expressed Pdgfrβ was significantly higher than the percentage expressing Pdgfrα, consistent with the higher proportion of Pdgfrβ+ mesenchymal cells at this early skin developmental stage (Gupta et al., 2019; Figure 5C).

At E18.5 the papillary and reticular dermal layers are forming. There were significantly more Ng2− Pdgfrα+ blood vessel associated cells in the reticular compared to the papillary dermis at this time point. However, there was no significant difference in the percentage of Ng2+ Pdgfrα+ double positive and Ng2+ Pdgfrα- populations between the two dermal layers (Figures 5D,E). Ng2+ Pdgfrβ+ populations showed no difference in distribution between the two dermal layers (Figures 5F,G). In addition, the percentage of Ng2+ Pdgfrα+ and Ng2+ Pdgfrβ+ double positive perivascular populations was very similar in both layers (both approximately 30% in the papillary layer; 20 and 15%, respectively, in the reticular layer, Figures 5E,G).

In summary these results show that Ng2+ perivascular populations within developing skin express Pdgfrα and β. As Ng2 is commonly referred to as a pericyte marker (Ozerdem et al., 2001; Ozerdem and Stallcup, 2004; Armulik et al., 2011), this strongly suggests a lineage relationship between Ng2+ pericytes and fibroblast populations.

To investigate whether Pdgfrα or Pdgfrβ expressing perivascular cells arise from Ng2+ progenitors we performed lineage tracing by crossing Ng2CreER with ROSA26-tdTomato mice. In control experiments we demonstrated that Cre was not expressed in the absence of Tamoxifen and identified the optimal Tamoxifen dose for high Ng2 labeling efficiency (Supplementary Figure 5).

Mice were treated with Tamoxifen at E18.5 because at this time point there is high Ng2 expression, and similar levels of Ng2+ Pdgfrα+ / Ng2+ Pdgfrβ+ cells in the papillary and reticular layers. Mice were culled at either P2 or P28 (Figure 5H). Ng2+ lineage cells were found on approximately 57% of blood vessel associated cells at P2 and approximately 18% at P28 (Figures 5I–K). Ng2CreER; tdTomato mice were co-stained with antibodies to Cd31 and either Pdgfrα or Pdgfrβ (Figures 5L–O). It is important to note that after Tamoxifen induction at E18.5 Ng2CreER^*t*^; tdTomato mice co-stained with Pdgfrα showed no tdTomato expression in interfollicular fibroblast subpopulations (Figure 5L and Supplementary Figure 6). Strong co-expression of Ng2 lineage positive cells and Pdgfrα was observed in approximately 54% of blood vessels in the papillary dermis and 64% in the reticular dermis (Figures 5L,N). Thus Ng2+ dermal cells give rise to Ng2+ Pdgfrα+ and Ng2+ Pdgfrα- perivascular populations. When skin from Ng2CreER^*t*^; tdTomato mice treated with Tamoxifen at E18.5 and collected at P28 was co-stained with anti-Pdgfrβ (Figure 5M) this revealed that 67% of Ng2Cre lineage perivascular cells expressed Pdgfrβ in the papillary dermis whilst 66% expressed Pdgfrβ in the reticular dermis (Figure 5O).

We conclude that Ng2 lineage cells do not contribute to interfollicular fibroblasts but instead are restricted to forming Ng2+ blood vessel associated cells, the majority of which express Pdgfrα and/or Pdgfrβ.

### Papillary and Reticular Fibroblast Lineages Contribute to Ng2+ Perivascular Populations

Next we investigated the contribution of specific dermal fibroblast subpopulations to Ng2+ perivascular populations using papillary (Lrig1+) and reticular (Dlk1+) fibroblast lineage markers previously identified to arise from a Pdgfrα progenitor at E12.5 (Driskell et al., 2013).

**FIGURE 6:**
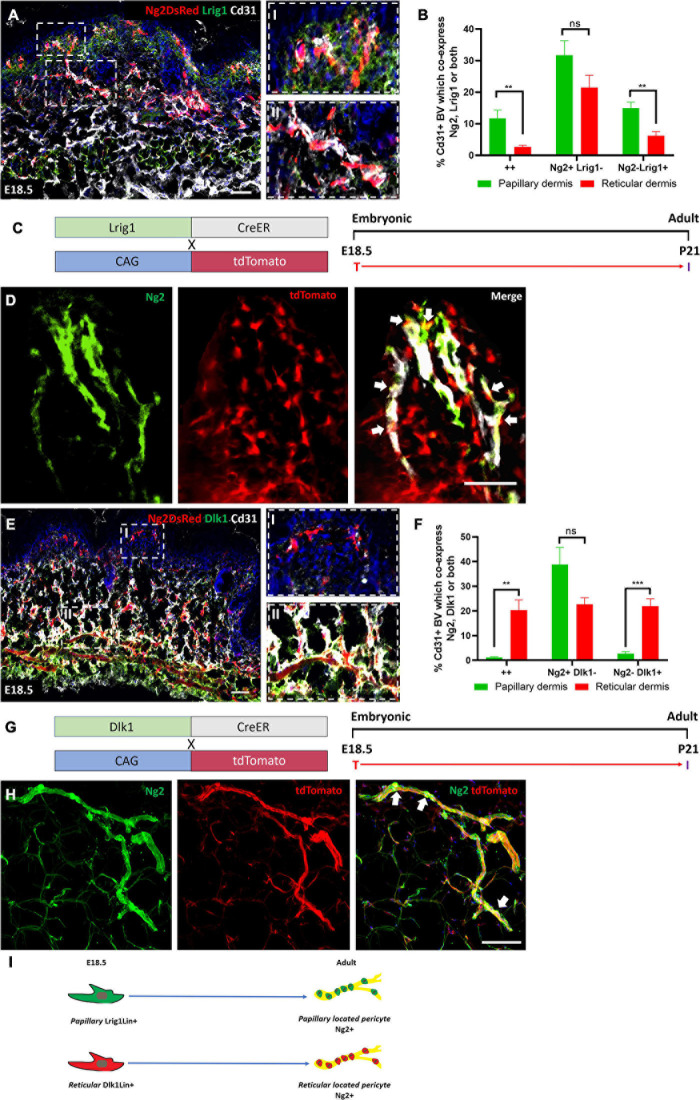
Papillary and reticular fibroblast lineages contribute to Ng2+ perivascular populations. **(A)** Ng2 DsRed mouse back skin at E18.5 labeled with Lrig1 (green) and Cd31 (gray). Higher magnified panels on the righthand side show papillary (I) and reticular blood vessels (II). **(B)** The percentage of Cd31+ blood vessels with Ng2 Lrig1 positive populations. **(C)** Lrig1 lineage tracing mouse lines and labeling strategy. **(D)** Representative immunostaining of Lrig1CreER; tdTomato P21 back skin labeled with Ng2 (green). White arrows highlight Lrig1Cre Ng2+ cells on the blood vessels. **(E)** Representative images of Ng2 DsRed mouse back skin at E18.5 labeled with Dlk1 (green) and Cd31 (gray). Higher magnification panels on the right-hand side show papillary (I) and reticular (II) blood vessels. **(F)** The percentage of Cd31+ blood vessels in the papillary or reticular layer of the dermis with Ng2 and Dlk1-positive cell populations. **(G)** Dlk1 lineage tracing: mouse lines and labeling strategy. **(H)** Representative immunostaining from Dlk1CreER; tdTomato back skin at P21 labeled with Ng2. White arrows highlight Dlk1Cre Ng2+ cells on the blood vessels. **(I)** Schematic demonstrating that in adult mouse back skin Lrig1 and Dlk1 fibroblast lineages contribute to Ng2+ perivascular populations in their respective dermal layers. **P≤0.01 and ****P≤0.0001. Scale bars 50 μm. Paired *T*-test. Error bars represent ±SD.

To determine the contribution of the papillary fibroblast lineage to Ng2+ perivascular populations we first assessed the expression of Lrig1 and Ng2 in Ng2DsRed mice at E18.5 (Figure 6A). Lrig1 is highly expressed by papillary fibroblast progenitors between E16.5 and P2. As expected, significantly more Ng2+ Lrig1+ double positive and Ng2− Lrig1+ perivascular cells were observed in the papillary layer compared to the reticular layer of the dermis (Figure 6B). Approximately 12% of perivascular cells were Ng2+ Lrig1+ double positive within the papillary dermis (Figure 6B). At P0 the percentage of perivascular cells that were Ng2+ Lrig1+ double positive was significantly lower than at E18.5 (Figures 6A,B and Supplementary Figures 7A,B). Therefore, lineage tracing of papillary fibroblasts was conducted using Lrig1CreER mice crossed with ROSA26-tdTomato mice from E18.5 until adulthood (P21) (Figure 6C and Supplementary Figure 8A) (Figure 6C). Immunostaining for Ng2 showed that approximately 40% (*N* = 7, ±SD: 13) of blood vessels in the papillary dermal layer had Lrig1Cre labeled Ng2+ associated cells (Figure 6D and Supplementary Figures 8B,C), demonstrating that a large portion of Ng2+ perivascular cells in the papillary dermis arose from the Lrig1+ papillary fibroblasts.

To determine the contribution of the reticular fibroblast lineage to Ng2+ perivascular populations Dlk1 expression was first assessed in Ng2DsRed developing back skin. Dlk1 is enriched in reticular fibroblast progenitors between E16.5 and P1 (Driskell et al., 2013). In line with these observations, in Ng2DsRed mouse back skin at E18.5 there were significantly more Ng2+ Dlk1+ double positive and Ng2− Dlk1+ perivascular cells in the reticular compared to the papillary layer of the dermis (Figures 6E,F). While at E18.5 approximately 20% of perivascular cells were Ng2+ Dlk1+ in the reticular dermis, this decreased to approximately 4% at P0 (Figure 6F and Supplementary Figures 7C,D). We therefore performed lineage tracing using Dlk1CreER mice crossed with ROSA26-tdTomato mice that were treated with Tamoxifen at E18.5 and traced the population until P21 (Figures 6G,H and Supplementary Figures 8D,E). At P21 approximately 84% (*N* = 6, ±SD: 3.52) of blood vessels in the reticular dermis had Dlk1CreER labeled Ng2+ cells (Figure 6H and Supplementary Figures 8E,F), demonstrating that the majority of Ng2+ perivascular cells in the reticular layer arose from the Dlk1+ reticular fibroblast lineage.

We conclude that a significant proportion of Ng2+ perivascular cells in adult mouse back skin originate from either the papillary or the reticular fibroblast lineage, according to which layer of the dermis the blood vessels are located (Figure 6I).

### During Wound Healing Ng2+ Dermal Populations Contribute Only to Blood Vessel Associated Cells

To investigate the contribution of Ng2+ cell populations to skin repair, we created full thickness 2 mm diameter circular wounds in adult back skin as previously described (Rognoni et al., 2016). We first examined wound healing in Ng2Dsred reporter mice, collecting skin at 4-, 7-, and 10-days post wounding (DPW) (Supplementary Figures 9A–C). Ng2+ perivascular cells could be visualized within wound beds at all-time points; however, the vascular networks observed at 10DPW in the papillary layer underneath the regenerated epidermis were more complete than at 4 and 7DPW (Supplementary Figures 9A–C). At all analyzed time points there was a similar percentage of blood vessel associated cells expressing Ng2 within and outside the wound bed, suggesting a similar distribution of Ng2+ perivascular populations within healthy and regenerating dermis (Supplementary Figure 9).

**FIGURE 7:**
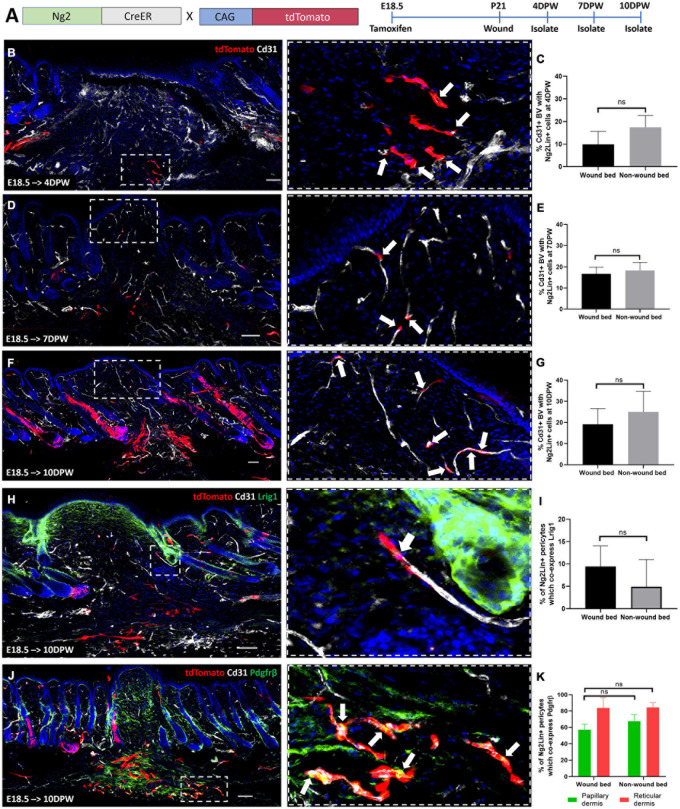
Ng2CreER labelled perivascular cells only contribute to the regeneration of blood vessel associated cells during wounding. **(A)** Ng2CreER^*t*^; tdTomato mouse line with labeling and wounding strategy. **(B–G)** Representative immunostaining of Ng2CreER^*t*^; tdTomato adult mouse back skin with Tamoxifen induction at E18.5, tracing Ng2+ lineage expression to 4 **(B)**, 7 **(D)**, and 10 **(F)** days post wounding, with Cd31 labeled blood vessels (gray) and DAPI nuclear counterstain (blue). Panels on the right-hand side are higher magnification images of boxed regions on the left-hand side. White arrows indicate Ng2 lineage positive blood vessel associated cells. **(C,E,G)** Graphs depict the percentage of Cd31 positive blood vessels within the wound bed and outside the wound bed, with Ng2CreER^*t*^ tdTomato labeled cells. **(H)** Representative immunostaining of Ng2CreER^*t*^; tdTomato adult mouse back skin with tamoxifen induction at E18.5 and isolation at 10 days post wounding labeled with Lrig1 (green) and Cd31 (gray). The higher magnification panel shows the presence of one Ng2 tdTomato+ Lrig1+ perivascular cell. **(I)** Graph depicting the percentage of Cd31 positive blood vessels inside and outside the wound bed with Ng2CreER^*t*^ tdTomato labeled perivascular cells expressing Lrig1. **(J)** Representative immunostaining of Ng2CreER^*t*^; tdTomato adult mouse back skin with tamoxifen induction at E18.5 isolated at 10 days post wounding labeled with Pdgfrβ (green) and Cd31 (gray). The higher magnification panel includes white arrows highlighting Ng2CreER^*t*^ tdTomato+ Pdgfrβ+ perivascular populations. **(K)** Graph depicting the percentage of Cd31 positive blood vessels with Ng2 tdTomato labeled Pdgfrβ positive perivascular cells present in papillary and reticular layers of the dermis inside and outside the wound bed. Images are representative of *N* = 3 mice per time point. Scale bars 100 or 50 μm (higher magnification views). Statistical test: Paired *T*-test. Error bars represent ±SD.

To understand how Ng2 labeled perivascular cells contribute to skin regeneration, Ng2CreER^*t*^; tdTomato skin treated with Tamoxifen at E18.5 was analyzed at 4, 7, and 10DPW (Figure 7A). At 4PW 10% of blood vessels had Ng2 lineage-positive associated cells (Figures 7B,C). This increased to 20% at 7 and 10DPW (Figures 7D–G), indicating that Ng2CreER^*t*^; tdTomato labeled cells were expanding during wound repair.

Co-staining with Lrig1 antibody revealed that no Ng2CreER^*t*^; tdTomato labeled cells contributed to interfollicular Lrig1+ dermal cells within healing wounds (Figure 7H). However, 9% of Ng2CreER^*t*^; tdTomato blood vessel associated cells co-expressed Lrig1 in the wound bed at 10DPW, compared to 6% in unwounded skin (Figure 7I).

To investigate whether Ng2 lineage cells gave rise to Pdgfrβ expressing blood vessel associated cells within a regenerating wound bed, tdTomato+ cells were co-stained with anti-Pdgfrβ at 10DPW (Figure 7J). This showed that the density of blood vessel associated Ng2CreER labeled Pdgfrβ+ cells was comparable within and outside the wound bed (Figures 7J,K).

In summary we found that Ng2+ perivascular cell heterogeneity is maintained during full thickness skin repair. In contrast to other perivascular cell populations (Dulauroy et al., 2012) Ng2 lineage cells did not contribute to the regeneration of dermal fibroblast populations.

### Papillary and Reticular Fibroblast Lineages Give Rise to Ng2+ Perivascular Cells During Wound Healing

**FIGURE 8:**
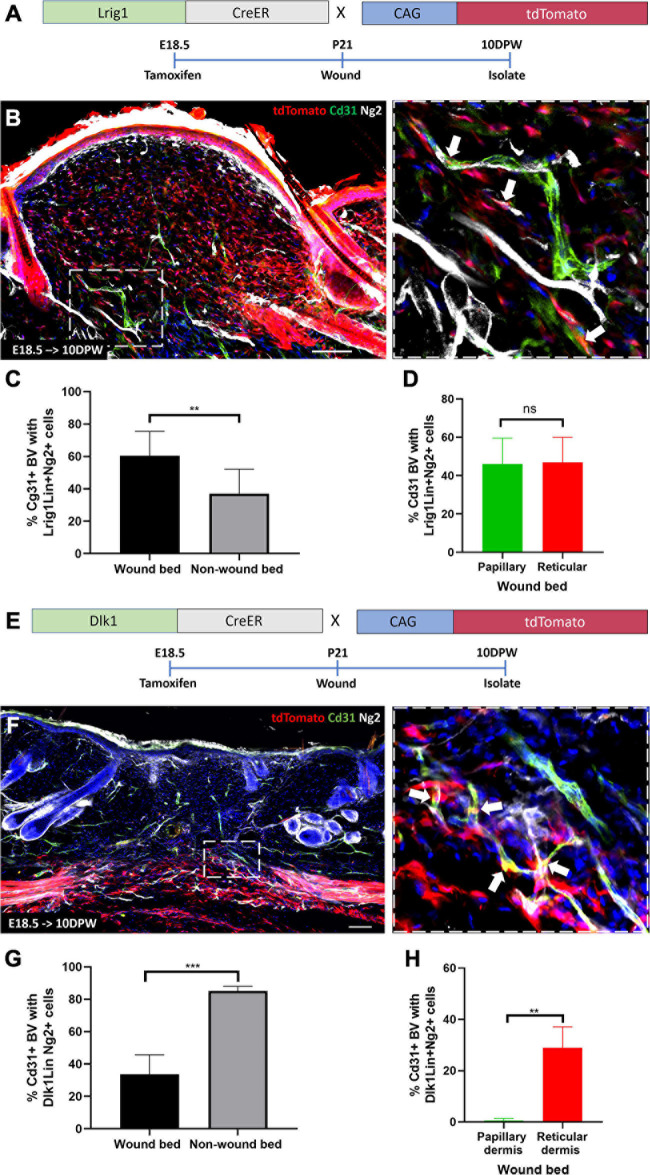
Papillary and reticular fibroblast lineages give rise to Ng2+ perivascular populations during wound healing. **(A)** Lrig1CreER; tdTomato mouse line with labeling and wounding strategy. **(B)** Representative immunofluorescence images of skin from Lrig1CreER; tdTomato mice treated with tamoxifen at E18.5 and isolated at 10 days post wounding labeled with Cd31 (green), Ng2 (gray) and DAPI nuclear counterstain (blue). The panel on the right-hand side is a higher magnification image of part of boxed region on the left-hand side. White arrows indicate Lrig1CreER tdTomato blood vessel resident perivascular cells expressing Ng2. **(C)** The percentage of Cd31 positive blood vessels within and outside the wound bed with Lrig1 tdTomato positive Ng2 expressing cells at 10 days post wounding. **(D)** Graph showing no significant difference in the percentage of Cd31 positive blood vessels with Lrig1CreER tdTomato and Ng2 expression in papillary and reticular layers of the regenerating wound bed dermis. **(E)** Dlk1CreER; tdTomato mouse line with labeling and wounding strategy. **(F)** Representative immunofluorescence images of Dlk1CreER; tdTomato lineage traced mouse back skin treated at E18.5 and isolated at 10 days post wounding labeled with Cd31 (green), Ng2 (gray) and DAPI nuclear counterstain (blue). The panel located on the right-hand side is a higher magnification image of part of the boxed region on the left-hand side. White arrows indicate Dlk1CreER tdTomato blood vessel resident cells expressing Ng2. **(G)** Graph depicting the percentage of blood vessels within and outside the wound bed with Dlk1 tdTomato Ng2 expressing cells. **(H)** Graph showing significantly higher percentage of Cd31 positive blood vessels with Dlk1 tdTomato and Ng2 expression in the reticular layer of the regenerating wound bed dermis when compared to the papillary layer. Statistical analysis was paired *T*-test. Error bars represent ±SD. *N* = 3 mice (3 sections per mouse) were quantified. Scale bars 100 or 50 μm (higher magnification views).

To investigate the contribution of specific fibroblast populations to Ng2+ perivascular regeneration in the wound bed, we injected Lrig1CreER; tdTomato and Dlk1CreER^*t*^; tdTomato mice with Tamoxifen at E18.5 and created 2 mm full thickness wounds in adult animals. Lrig1CreER tdTomato labeled cells were found throughout the dermis of the regenerating wound bed at 10DPW (Figures 8A,B). 60% of blood vessels within the wound bed had associated Lrig1Cre tdTomato+ Ng2+ expressing cells, compared to approximately 40% outside the wound bed (Figures 8A–C). In contrast to healthy back skin, where Lrig1Cre tdTomato Ng2+ perivascular cells were spatially restricted to the papillary layer, no distinct organization was maintained in regenerated skin (Figure 8D). This is in line with our previous observation that upper fibroblast lineages are redistributed within the entire wound bed during regeneration (Rognoni et al., 2018).

The Dlk1 lineage was confined to the reticular layer of the 10DPW wound bed and 35% of blood vessels contained Dlk1 tdTomato labeled Ng2+ perivascular cells, significantly less than those present outside the wound bed (Figures 8E–H). Thus, in a similar manner to unwounded tissue, the Dlk1 lineage contribution to Ng2+ perivascular cells was spatially restricted, albeit being much lower in abundance at 10DPW than in non-wounded skin (Figures 8F–H).

**FIGURE 9:**
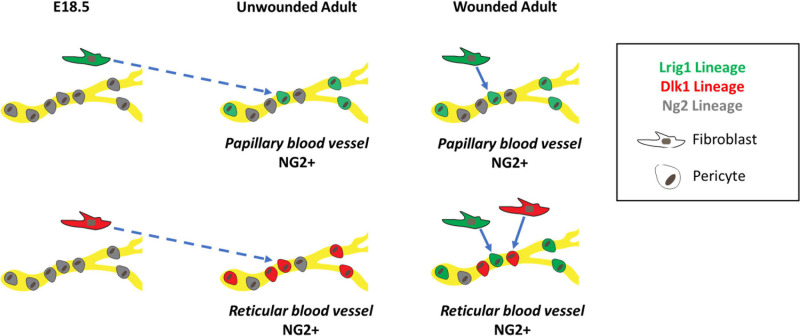
Schematic summary showing origins of Ng2+ skin pericytes in unwounded and wounded skin. In unwounded skin, Lrig1 (green), and Ng2 (gray) lineages, labeled at E18.5, give rise to Ng2+ blood vessel resident pericytes in the papillary layer of the dermis whilst Dlk1 (red) and Ng2 lineages give rise to Ng2+ blood vessel resident pericytes in the reticular layer of the dermis. These NG2+ pericytes can differ in their Pdgfr status. In unwounded skin Lrig1 and Dlk1 fibroblast lineages contribute to Ng2+ pericyte populations in a spatially restricted manner. However, in wounded skin, the Lrig1 and NG2 lineages can contribute to pericytes in both layers of the dermis whilst the contribution of the Dlk1 lineage remains spatially restricted to the reticular layer.

We conclude that in full thickness wounds both papillary and reticular fibroblasts contribute to Ng2+ perivascular cell regeneration, recapitulating the generation of the perivascular cell network during skin development (Figure 9).

## Discussion

We have found that Ng2+ perivascular cells represent a heterogeneous population of pericytes that are primarily derived from the papillary or reticular fibroblast lineages during development and skin wound repair (Figure 9), and do not contribute to non-vessel associated skin fibroblasts. In adult skin Ng2+ perivascular cells could be divided into four distinct populations: Ng2+ Pdgfrα+ Pdgfrβ+, Ng2+ Pdgfrα+ Pdgfrβ−, Ng2+ Pdgfrα− Pdgfrβ+ and Ng2+ Pdgfrα− Pdgfrβ− cells. The existence of multiple Ng2+ pericyte populations and the differences in their abundance in papillary and reticular dermis could reflect functional differences that warrant further investigation. As a precedent for this, there are differences in Wnt/β-catenin signaling in intradermal fibroblasts in the papillary and reticular layers (Rognoni et al., 2016).

While the functional role of Pdgfrα signaling in pericytes is unclear, Pdgfrβ expression plays a role in expansion and migration of pericytes along growing vessels (Armulik et al., 2005). Pericyte heterogeneity has also been described in other organs (Birbrair et al., 2013b; Barron et al., 2016). In lung two pericyte populations arise from the same FoxD1 progenitor, one of which co-expresses fibroblast and pericyte markers (Col1+ Pdgfrα+ Pdgfrβ+ Ng2+) (Barron et al., 2016). In skeletal muscle type 1 pericytes remain in the interstitial space, produce collagen and contribute to fibrosis whereas type 2 communicate with endothelial cells to form new vessels in healthy and tumor tissue (Birbrair et al., 2013a,b, 2014a, 2014b). So far single cell RNA sequencing of cells from adult human skin has identified one pericyte population, which expresses RGS5 and ACTA2 (Philippeos et al., 2018; Vorstandlechner et al., 2020). However, a second pericyte population has been identified in skin from patients with atopic dermatitis and psoriasis; these pericytes have a proposed role in leukocyte recruitment and TNFα-mediated signaling (Reynolds et al., 2020, 2021).

In the skin Ng2 expression is not restricted to pericytes, since it is also expressed by subsets of keratinocytes and Schwann cells (Sennett et al., 2015). Lineage tracing showed that while Ng2+ cells gave rise to multiple cell types they did not give rise to any dermal fibroblast subpopulations, including the APM, dermal papilla, DWAT, papillary and reticular layers. Ng2-lineage pericytes remained associated with blood vessels, giving rise to both Ng2+ Pdgfrα+ and Ng2+ Pdgfrβ+ populations, even during wound repair (Figure 9).

In our study we found that Ng2+ perivascular cells were primarily derived from interfollicular papillary and reticular fibroblasts during skin development and regeneration. Lrig1Cre and Dlk1Cre E18.5 labeled fibroblasts, corresponding to papillary and reticular fibroblast lineages, respectively, contributed to adult Ng2+ perivascular populations in a predominantly location dependent manner. This is in good agreement with the relationship between spatial location and lineage identity in dermal fibroblasts (Driskell et al., 2013; Korosec et al., 2019).

Since there is extensive evidence that Ng2 is highly expressed by pericytes (Ozerdem et al., 2001; Ozerdem and Stallcup, 2004; Armulik et al., 2011; Ieronimakis et al., 2013), the identification of Ng2+ perivascular cells arising from distinct fibroblast lineages reveals a previously unrecognized relationship between fibroblasts and pericytes during skin development. Adventitial fibroblasts and pericytes are both present in the adventitia layer of the blood vessel wall separated by the basal lamina (Kuwabara and Tallquist, 2017; Riew et al., 2018; Tinajero and Gotlieb, 2020) and share expression of several markers including Pdgfrα, Pdgfrβ, Nestin, Vimentin and Col1A1 (Lin et al., 2008; Riew et al., 2018; Jiang et al., 2021). However, adventitial fibroblasts do not express Ng2 (Ieronimakis et al., 2013; Jiang et al., 2021) and although pericytes and fibroblasts are both reported to differentiate into adipocytes (Tang et al., 2008; Cattaneo et al., 2020) we did not observe Ng2 progenitor derived adipocytes in the DWAT skin layer. Co-expression of Ng2 and Pdgfrα by blood vessel associated cells at E12.5 suggests that some Ng2+ pericytes originate from the Pdgfrα+ multipotent fibroblast progenitor described previously (Driskell et al., 2013). However, further studies are required to discover whether there are additional pericyte progenitors, for example cells that express Pdgfrβ and not Pdgfrα at E12.5. It was shown previously that approximately 13% of Ng2+ pericytes in skin arise from the myeloid lineage (Yamazaki et al., 2017). This is consistent with our data indicating that the majority but not all Ng2+ pericyte populations arise from interfollicular fibroblast lineages.

Lineage tracing showed that following wounding Ng2 lineage pericytes were unable to regenerate interfollicular fibroblasts in the skin. This contrasts with other organs in which pericytes can differentiate into myofibroblasts and contribute extensively to tissue repair (Humphreys et al., 2010; Chen et al., 2011). It may reflect a high degree of maturity of Ng2+ skin pericytes (Song et al., 2005; Morikawa and Ezaki, 2011; Stapor et al., 2014). Pericyte function may also be influenced by the organ in which the cells reside, as for example Type 1 pericytes (Nestin- Ng2+) contribute to fibrosis in skeletal muscle but not in the kidney or the heart (Birbrair et al., 2014a). Furthermore, injury type may influence pericyte behavior. In our study the wounds were only 2 mm in diameter, and previous studies have shown that wound size influences a range of responses, including papillary fibroblast regeneration, *de novo* hair follicle formation and epidermal Wnt signaling (Ito et al., 2007; Driskell et al., 2013; Rognoni et al., 2016).

At 10 days post wounding Dlk1Cre labeled Ng2 positive pericytes were associated with the new vessels of the lower dermis, although to a smaller degree than in undamaged skin. This may be a direct result of the increased contribution to Ng2+ pericytes from the Lrig1 lineage, which unlike in unwounded skin were not restricted to the papillary layer (Figure 9). The increased contribution of the Lrig1 lineage is also seen in wound bed fibroblasts (Driskell et al., 2013). Previous studies have shown that Wnt/ β-catenin signaling and TGFβ signaling influence fibroblast regeneration during wound repair (Dulauroy et al., 2012; Driskell et al., 2013; Marangoni et al., 2015; Plikus et al., 2017) and these signaling pathways may also determine the proportion of wound bed pericytes of different lineages.

## Conclusion

In conclusion, our studies have revealed a previously unknown degree of pericyte heterogeneity in mouse skin. We have demonstrated that the origin of Ng2+ pericytes is mainly determined by their dermal location—those in the upper dermis arise from the papillary fibroblast lineage, while those in the lower dermis originate from the reticular lineage, both during normal development and during wound healing. It remains to be seen whether different pericyte lineages have different functions and, if so, whether differences in Wnt, Hh, and TGF-β signaling in different dermal layers are involved (Lichtenberger et al., 2016; Mastrogiannaki et al., 2016). Further studies have the potential to increase our understanding of skin development and regeneration, and to uncover novel strategies to improve wound repair.

## Data Availability Statement

The original contributions presented in the study are included in the article/Supplementary Material, further inquiries can be directed to the corresponding author/s.

## Ethics Statement

The animal study was reviewed and approved by the King’s College London AWERB.

## Author Contributions

GG, ER, and FW: conceptualization, funding acquisition, and writing—review and editing. GG: data curation. GG, ER, and VS: formal analysis and visualization. GG and ER: investigation and methodology. ER and FW: project administration and supervision. GG and FW: writing—original draft. All authors contributed to the article and approved the submitted version.

## Conflict of Interest

FW is currently on secondment as Executive Chair of the UK Medical Research Council. The remaining authors declare that the research was conducted in the absence of any commercial or financial relationships that could be construed as a potential conflict of interest.
