# Supplementary material for: Distinct Fibroblast Lineages Give Rise to NG2+ Pericyte Populations in Mouse Skin Development and Repair
Source: Front Cell Dev Biol. 2021 May 28;9:675080. doi: 10.3389/fcell.2021.675080 (PMC8194079; doi:10.3389/fcell.2021.675080)
Supplement: Supplementary file 1 [file Table_1.pdf]

SUPPLEMENTARY MATERIAL.

SUPPLEMENTARY FIGURE LEGENDS

Supplementary Figure 1: Co-expression of Ng2DsRed and Ng2 in perivascular cells within mouse back skin. A-C: Immunostaining of Ng2 DsRed mice labelled with an antibody to Ng2 at P3 (**A**), P6 (**B**), and P36 (**C**). Representative images shown. **D**: ImageJ Manders Split Co-localisation Coefficient analysis was carried out to compare the co-expression of Ng2 protein (detected by antibody staining). N=3 mice at each developmental stage, 3 sections per mouse. Scale bars 50µm.

Supplementary Figure 2: Expression of Ng2 in postnatal mouse back skin. Mouse back skin from Ng2DsRed mice labelled at P0 **(A),** P3 **(B),** P21 **(C)** and P50 **(D).** Sections were immunolabelled with αSMA (green) and Cd31 (grey) and counterstained with DAPI (blue) to detect nuclei. Representative images shown. **A:** Note Ng2 positive cells labelling small blood vessels associated with hair follicles and wrapping around large blood vessels in the lower dermis**. B:** Note Ng2 positive cells labelling small blood vessels throughout the dermis and Ng2+ expression within hair follicle dermal sheath cells**. C:** Ng2 positive cells can be seen at the leading front of blood vessels in the lower dermis. Ng2 is also expressed by cells in the lower proximal cup of hair follicles and encircling the hair follicle bulge. **D:** Ng2 positive cells can be seen labelling blood vessels in the lower layer of the dermis. Ng2 expression is also detected by cells in the lower proximal cup of hair follicles and encircling the hair follicle bulge adjacent to the APM. N=3. Scale bar 100µm.

Supplementary Figure 3: Ng2 expression by non-blood vessel associated cells in adult back skin. Ng2DsRed mouse skin was immunolabelled with: (**A**) anti-αSMA to indicate the arrector pili muscle (APM) (arrows highlight co-expression of αSMA and Ng2); (**B, C)** Keratin 14 (KRT14) to indicate epithelial cells of the hair follicle. Hair follicle dermal sheath is shown in **B** and lower proximal cup in **C**. Sections were counterstained with DAPI (blue) to detect cell nuclei. All micrographs in a single panel show the same field. Images shown are representative of N=3 mice. Scale bars 50µm.

Supplementary Figure 4: Ng2 expressing cells encircle the hair follicle bulge adjacent to the arrector pili muscle (APM). A, B: Ng2 DsRed mouse back skin (P50) labelled with antibodies to Nephronectin (**A**) and Egfl6 (**B**). Right hand panels are higher magnification views of boxed areas in left hand panels. Areas of Ng2 and nephronectin co-expression at the anchor point of the APM to the bulge are highlighted by arrows in **A**. Areas of Ng2 and Egfl6 co-expression in the bulge are highlighted by arrows in **B**. Images shown are representative of N=3 mice. Scale bars 100µm.

Supplementary Figure 5: Determining optimal Tamoxifen dosing regimen for Ng2CreER. A: Ng2CreER^{t}; tdTomato mouse line with tamoxifen labelling and isolation strategy. **B**: Ng2CreER^{t};

tdTomato mice were injected with Tamoxifen at P31 and back skin was isolated at P50. Ng2CreER^t tdTomato expression was primarily detected by cells within the hair follicle bulge. **C:** Ng2CreER^t; tdTomato mice were injected with corn oil, as a vehicle control, at P31 and back skin was isolated at P50. No Ng2 tdTomato expression was detected. **D**: Ng2CreER^t**;** tdTomato mice tamoxifen dose strategy. Panels which correspond to each dose regimen are highlighted. **E**: Mice were administered with one intraperitoneal injection of Tamoxifen at P46 and back skin was isolated at P50. **F**: Ng2CreER^t; tdTomato mice were administered with 3 intraperitoneal injections of Tamoxifen 24hours apart at P46, P47, P48, and back skin was isolated at P50. No difference in tdTomato expression was observed between the two dosing regimens. Sections were counterstained with DAPI (blue). Mice were littermates, N=3 per condition. Images shown are representative of N=3 mice. Scale bars 100µm.

Supplementary Figure 6: Ng2 tdTomato lineage positive populations do not contribute to interfollicular dermal fibroblast populations. A: Ng2CreER^t; tdTomato mouse line and tamoxifen induction and isolation strategy. **B:** Immunofluorescence images from Ng2CreER^t; tdTomato mice labelled with tamoxifen at E18.5 and isolated at P2 labelled with Pdgfrα (green). No co-expression of between Ng2CreER^t tdTomato and Pdgfrα was detected, apart from in cells associated with blood vessels. All micrographs in a single panel show the same field. Merged images are counterstained with DAPI (blue). Representative images shown. N=3, 3 sections per mouse. Scale bars 100µm.

Supplementary Figure 7: Few Ng2+ perivascular cells express Lrig1 or Dlk1 at P0. **A, C**: Immunostaining of Ng2DsRed P0 mice labelled with Lrig1 (green) (**A**) or Dlk1 (green) (**C**) and Cd31 (grey) with DAPI nuclear counterstain (blue). Although Dlk1 is expressed by blood vessel cells (**C**), most are adjacent to Ng2+ cells rather than co-expressed by Ng2+ cells. **B, D:** Quantification of average percentage of Lrig1+Ng2+ (**B**) and Dlk1+Ng2+ (**D**) double positive cells on papillary (**B**) and reticular (**D**) blood vessels. N=5 biological replicates 3 sections per mouse were analysed. Images shown are representative. Scale bar 50µm.

Supplementary Figure 8: E18.5 tamoxifen labelling of Lrig1 and Dlk1 CreER; tdTomato mice results in tdTomato expression enriched in either the papillary or reticular layer of the dermis respectively. A: Lrig1 lineage tracing mouse lines and labelling strategy. **B:** Immunostaining from Lrig1CreER; tdTomato P21 back skin labelled with Cd31. Sperate panels show single channel images of the same field of view. tdTomato expression is specific to the papillary layer of the dermis. **C**: Graph depicting the percentage of Cd31 labelled blood vessels with Lrig1CreER tdTomato positive cells in the papillary and reticular dermis at P21. **D**: Dlk1 lineage tracing mouse line and labelling strategy. **E**: Immunostaining from Dlk1CreER; tdTomato P21 back skin labelled with Cd31. Sperate panels show single channel images of the same field of view. TdTomato expression is specific to the reticular layer

of the dermis. **F**: Graph depicting the percentage of Cd31 labelled blood vessels with Dlk1CreER tdTomato positive cells in the papillary and reticular dermis at P21. Statistical analysis paired T Test. Error bars represent ±SD. Scale bars 100µm. Representative images shown. N=3.

Supplementary Figure 9: Ng2 DsRed regenerating wound beds. A-C: Immunostaining of Ng2 DsRed adult mouse back skin at 4 (**A**), 7 (**B**), and 10 (**C**) DPW with Cd31 (grey) and DAPI nuclear counterstain (blue). Panels on the right-hand side are higher magnification images of boxed regions on the left-hand side. Arrows indicate Ng2 expression by blood vessel resident cells. Graphs depict the percentage of blood vessels within and outside the wound bed with Ng2 expressing cells. N=3, 3 sections per mouse. Scale bars 100µm in large image and 50µm in higher magnified micrographs. Representative images shown. Error bars ±SD.

SUPPLEMENTARY TABLE

Supplementary Table 1: Antibodies used for immunofluorescence staining

**Antibodies**	**Species**	**Dilution**	**Supplier**
Tbx18	Rabbit	1:500	Abcam (Ab115262)
Nestin	Rabbit	1:500	Sigma Aldrich (N5413-100UG)
Pdgfrβ	Rabbit	1:1000	Abcam (Ab32570)
Ng2	Rabbit	1:1000	Merk (Ab5320)
Pdgfrα	Rabbit	1:500	Cell Signalling (3174s)
Lrig1	Goat	1:250	R&D Systems (AF3688)
Dlk1	Goat	1:250	R&D Systems (AF1144)
Cd31	Rat	1:500	BD Pharming (553370)
