# Supplementary figures and images for: Distinct Fibroblast Lineages Give Rise to NG2+ Pericyte Populations in Mouse Skin Development and Repair
Source: Front Cell Dev Biol. 2021 May 28;9:675080. doi: 10.3389/fcell.2021.675080 (PMC8194079; doi:10.3389/fcell.2021.675080)

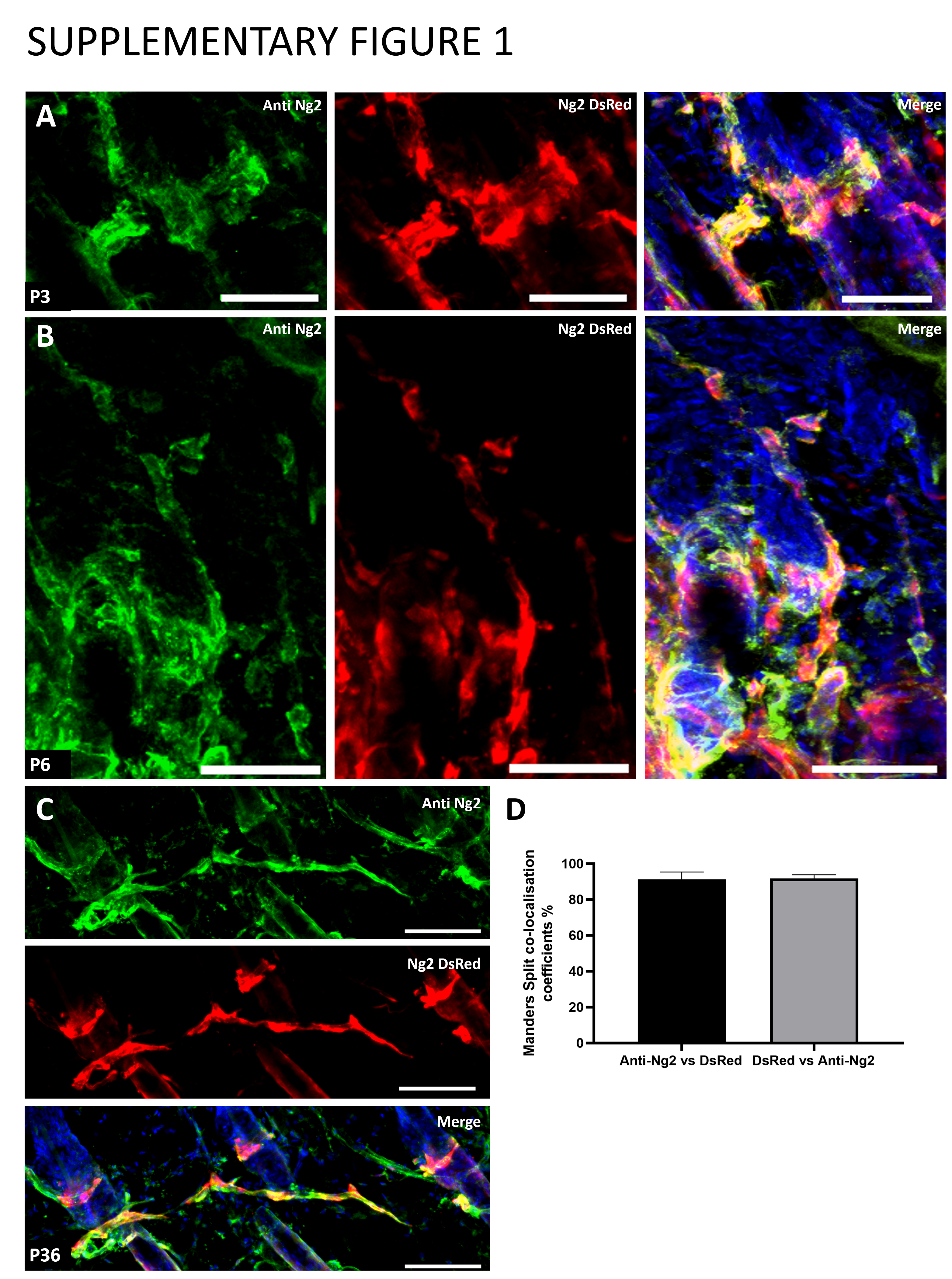

Supplement: Supplementary file 2 [file Image_1.TIF]

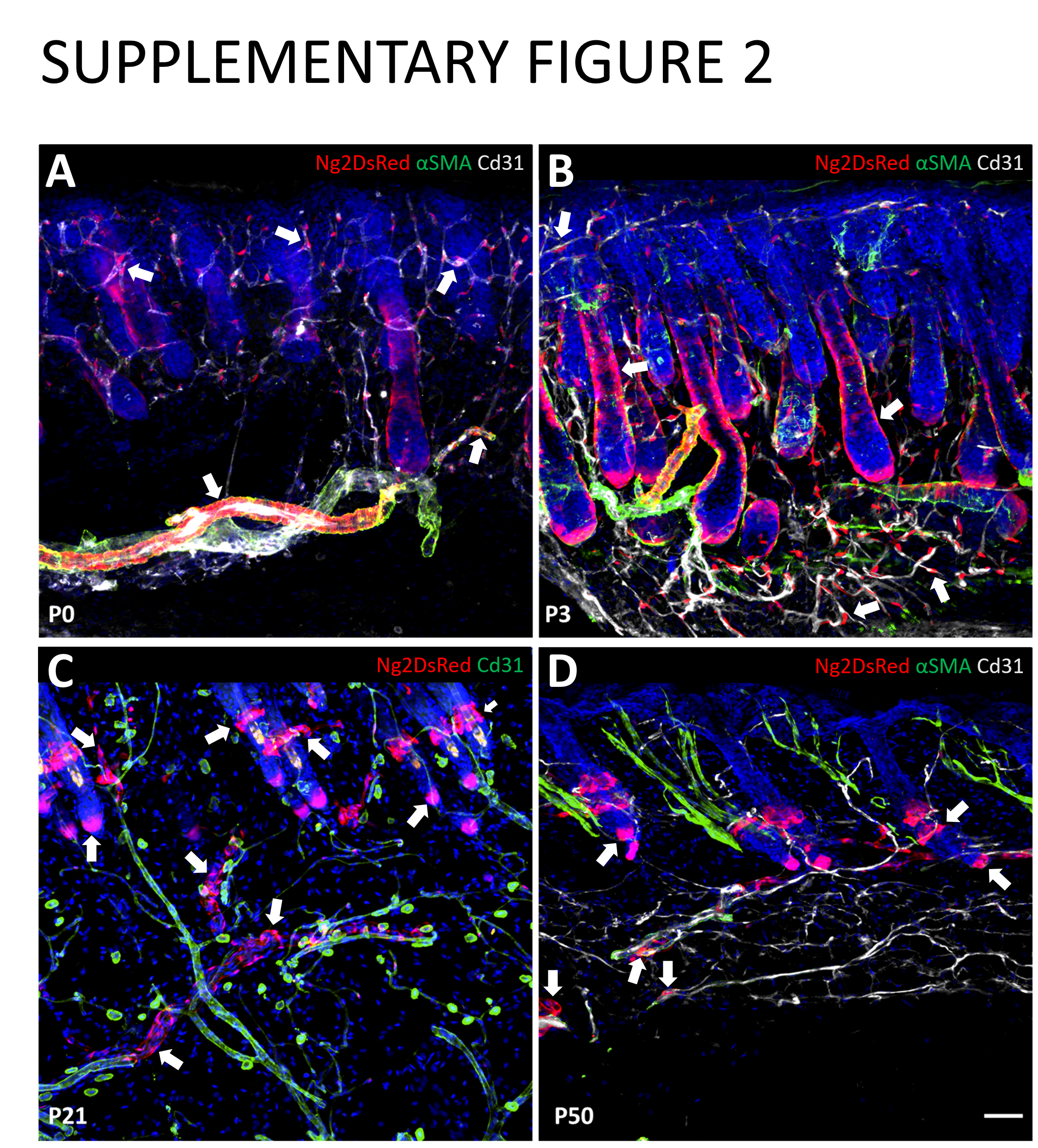

Supplement: Supplementary file 3 [file Image_2.TIF]

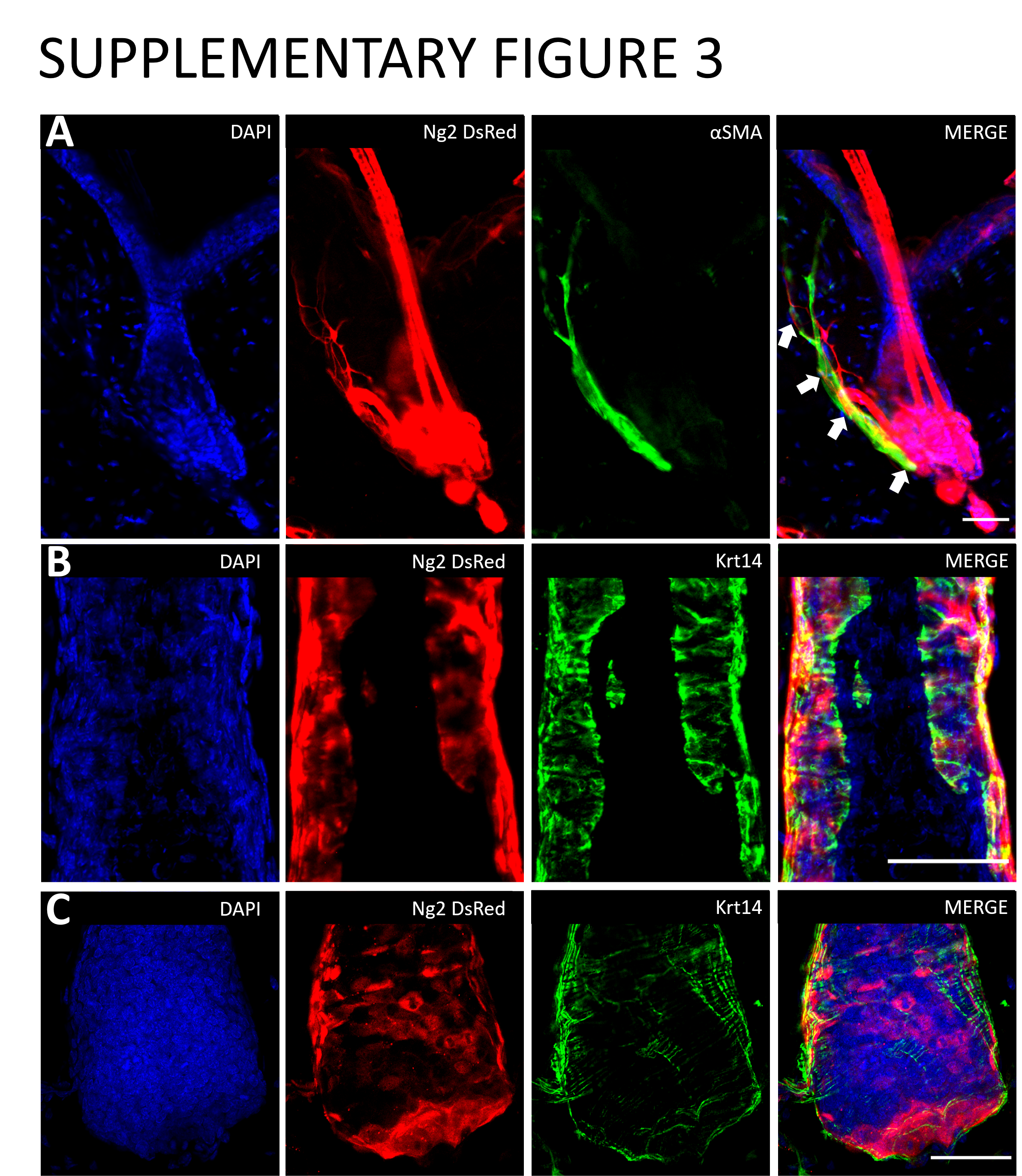

Supplement: Supplementary file 4 [file Image_3.TIF]

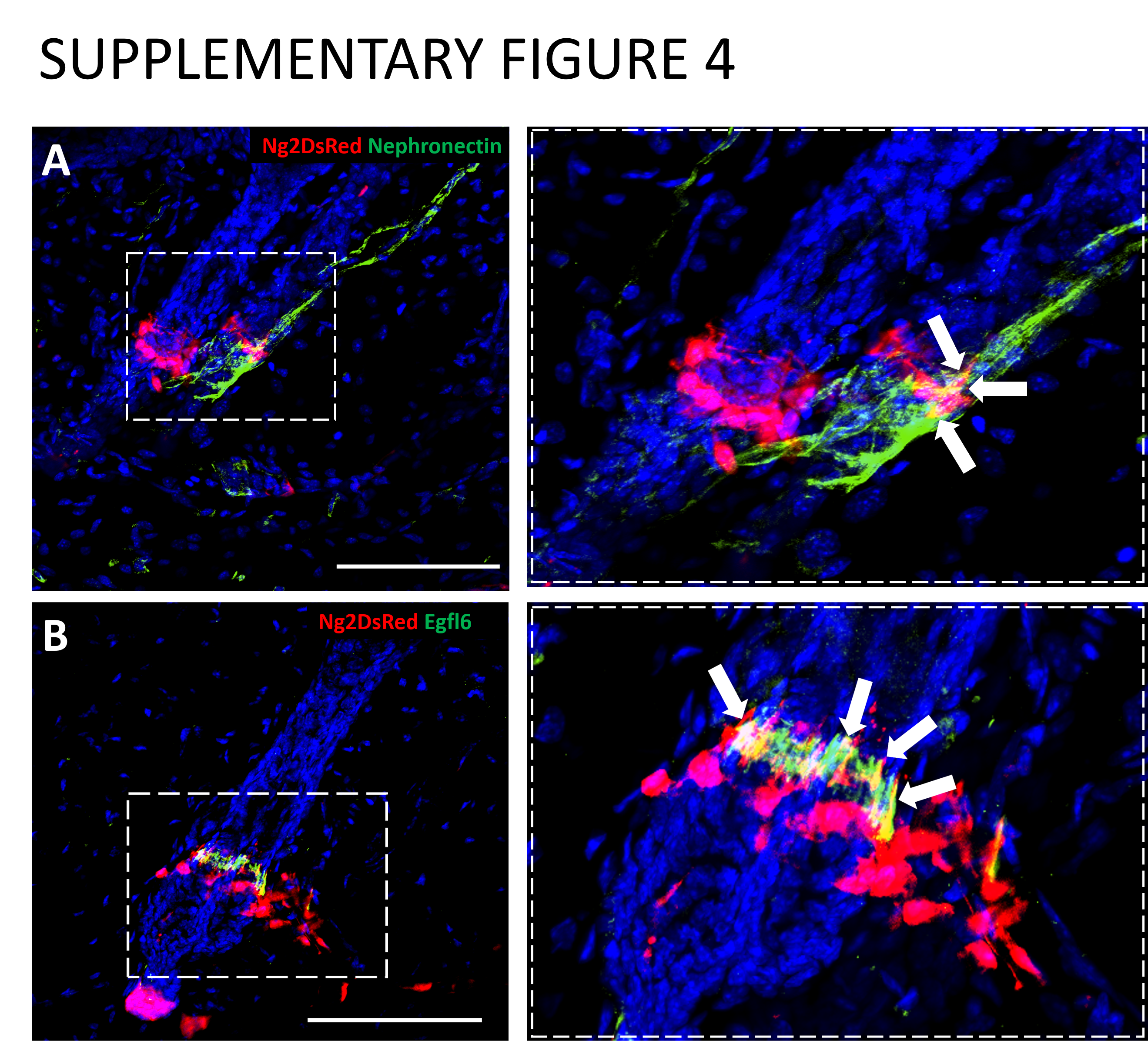

Supplement: Supplementary file 5 [file Image_4.TIF]

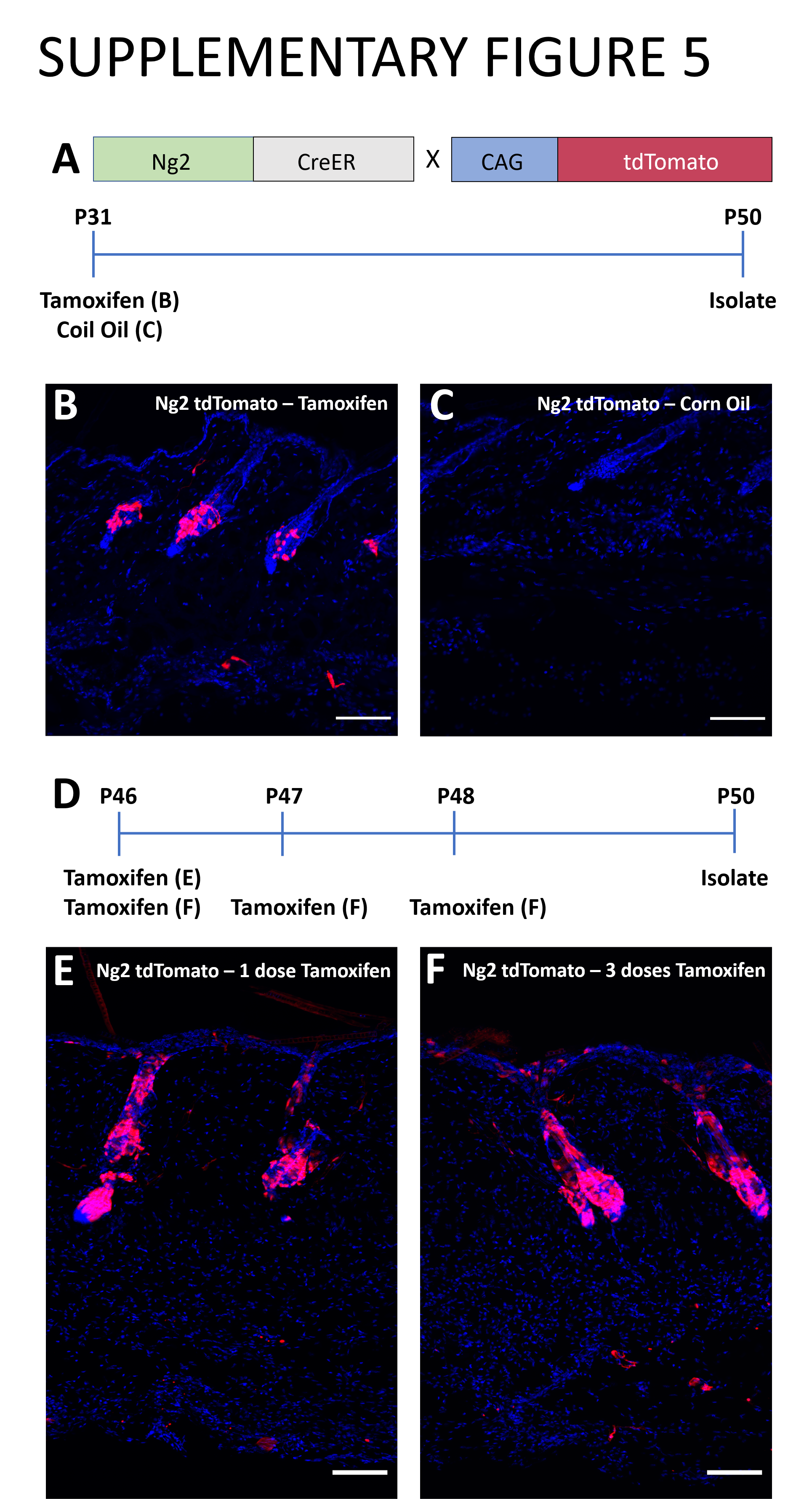

Supplement: Supplementary file 6 [file Image_5.TIF]

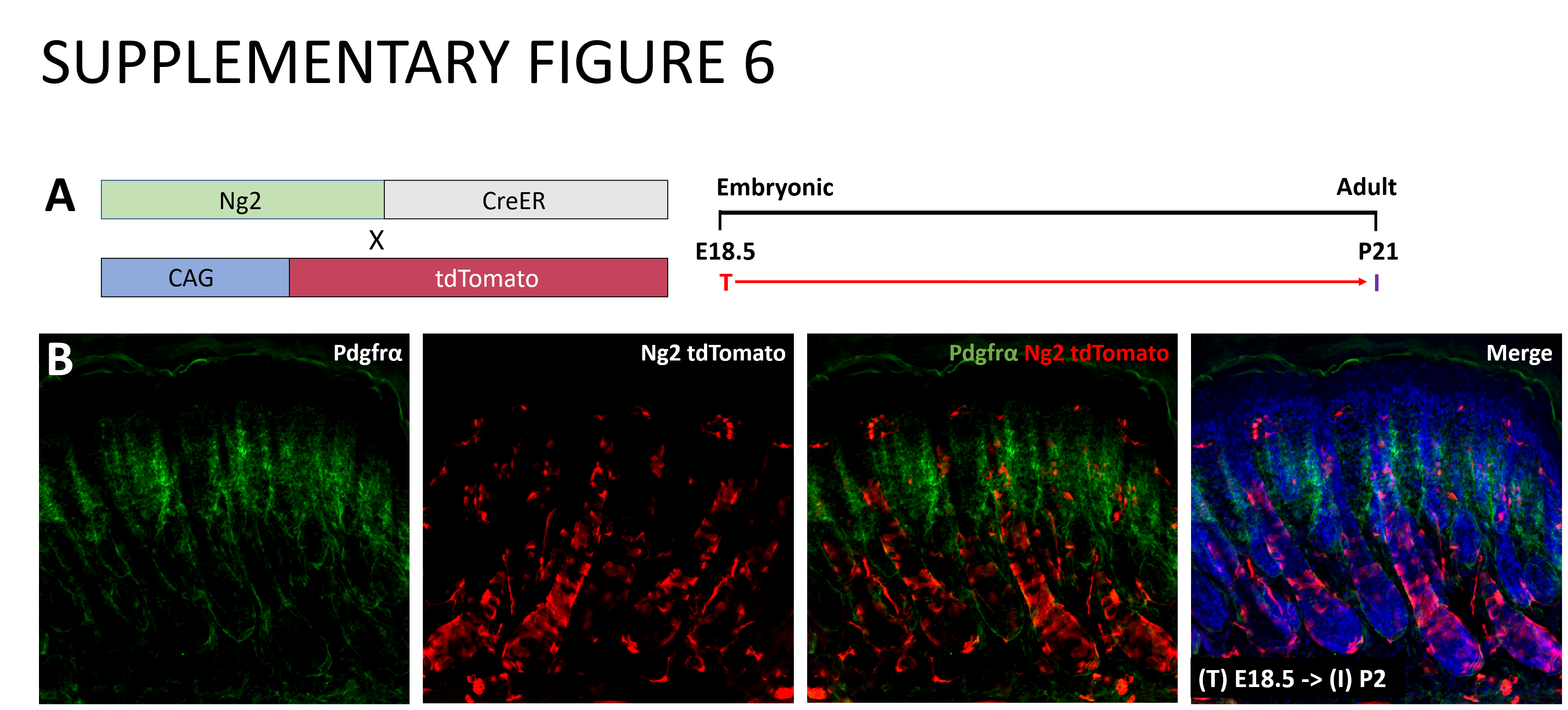

Supplement: Supplementary file 7 [file Image_6.TIF]

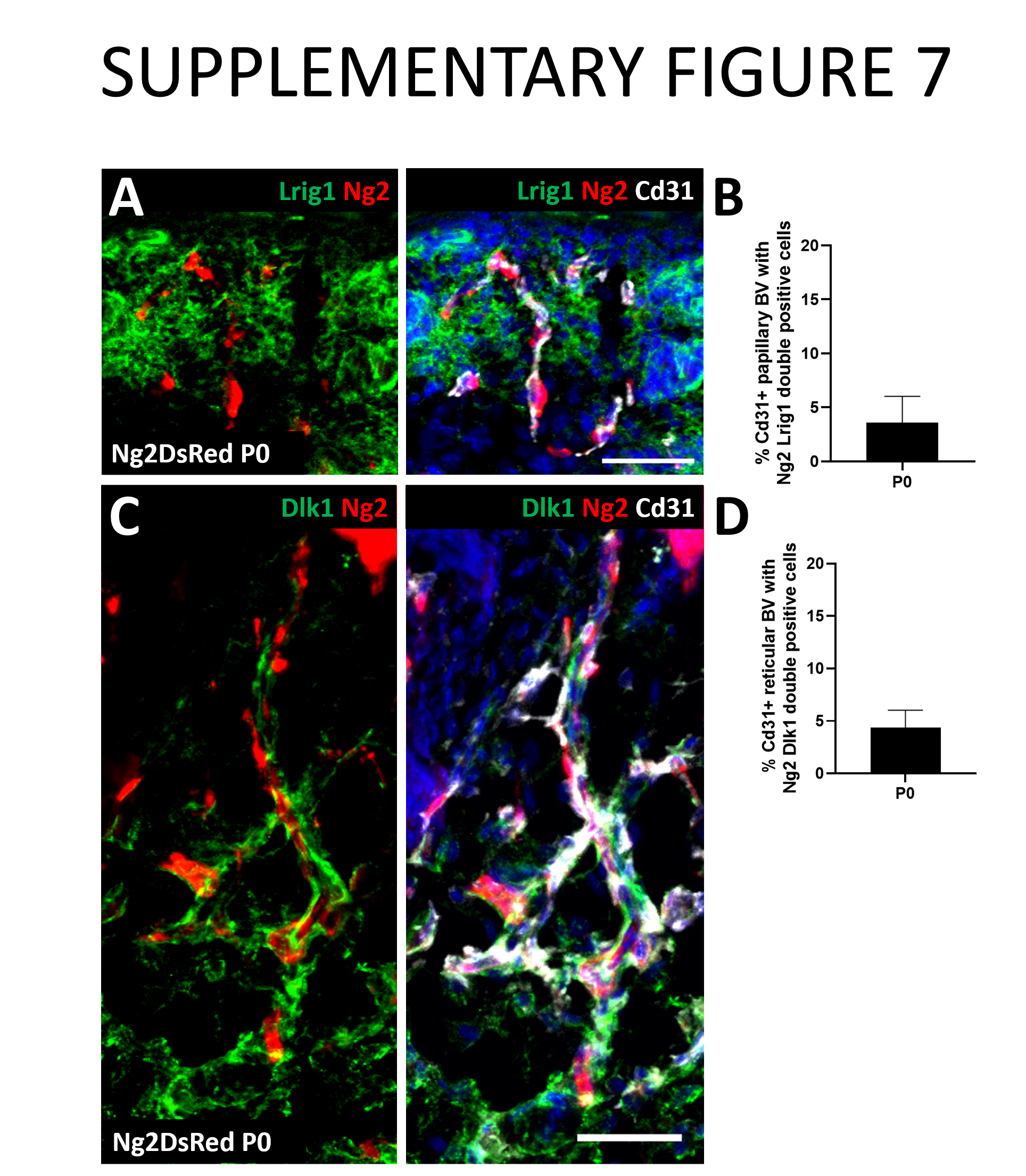

Supplement: Supplementary file 8 [file Image_7.TIF]

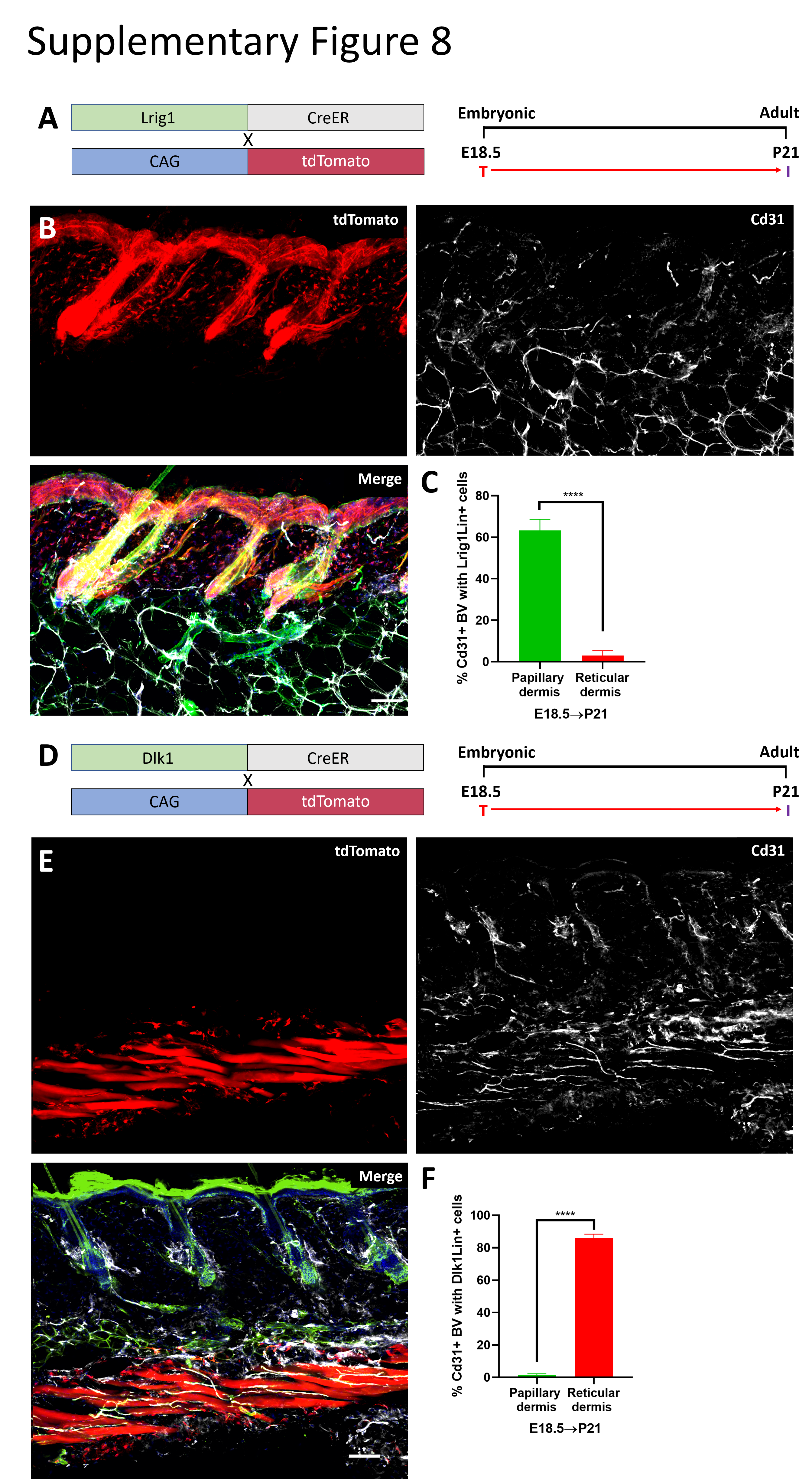

Supplement: Supplementary file 9 [file Image_8.TIF]

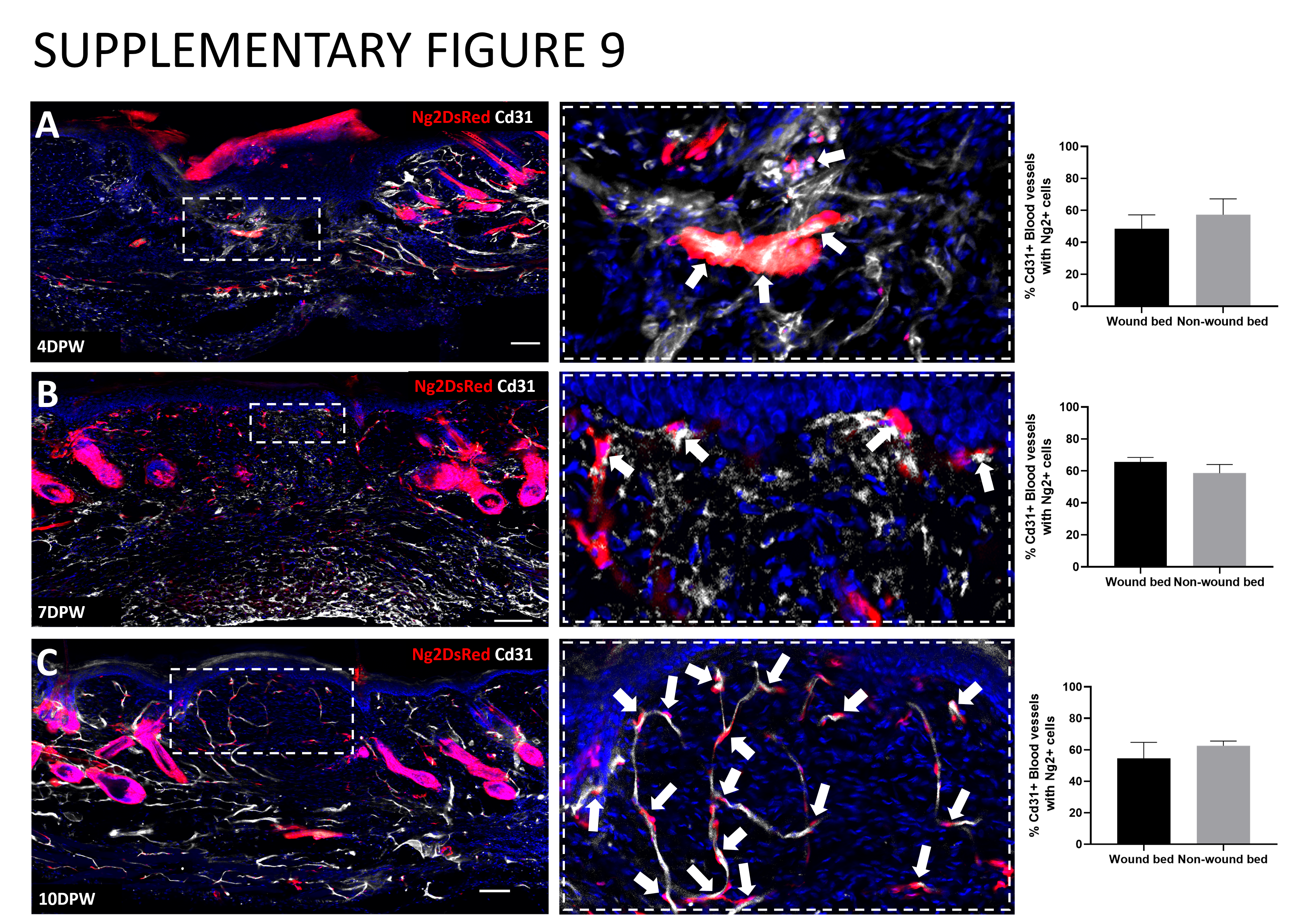

Supplement: Supplementary file 10 [file Image_9.TIF]
